# Attention controls multisensory perception via two distinct mechanisms at different levels of the cortical hierarchy

**DOI:** 10.1371/journal.pbio.3001465

**Published:** 2021-11-18

**Authors:** Ambra Ferrari, Uta Noppeney

**Affiliations:** 1 Computational Neuroscience and Cognitive Robotics Centre, University of Birmingham, Birmingham, United Kingdom; 2 Donders Institute for Brain, Cognition and Behaviour, Radboud University, Nijmegen, The Netherlands; Oxford University, UNITED KINGDOM

## Abstract

To form a percept of the multisensory world, the brain needs to integrate signals from common sources weighted by their reliabilities and segregate those from independent sources. Previously, we have shown that anterior parietal cortices combine sensory signals into representations that take into account the signals’ causal structure (i.e., common versus independent sources) and their sensory reliabilities as predicted by Bayesian causal inference. The current study asks to what extent and how attentional mechanisms can actively control how sensory signals are combined for perceptual inference. In a pre- and postcueing paradigm, we presented observers with audiovisual signals at variable spatial disparities. Observers were precued to attend to auditory or visual modalities prior to stimulus presentation and postcued to report their perceived auditory or visual location. Combining psychophysics, functional magnetic resonance imaging (fMRI), and Bayesian modelling, we demonstrate that the brain moulds multisensory inference via two distinct mechanisms. Prestimulus attention to vision enhances the reliability and influence of visual inputs on spatial representations in visual and posterior parietal cortices. Poststimulus report determines how parietal cortices flexibly combine sensory estimates into spatial representations consistent with Bayesian causal inference. Our results show that distinct neural mechanisms control how signals are combined for perceptual inference at different levels of the cortical hierarchy.

## Abstract 2

A combination of psychophysics, computational modelling and fMRI reveals novel insights into how the brain controls the binding of information across the senses, such as the voice and lip movements of a speaker.

## Introduction

In a busy restaurant, our senses are inundated with numerous diverse signals: talking voices, clinking glasses, and the sight and smell of food. To form a reliable percept, the brain needs to integrate sensory signals that come from common sources weighted by their relative reliabilities, giving a stronger weight to the more reliable signal [1–6]. Ample evidence has shown that multisensory integration is not only determined by the signals’ sensory reliabilities, but also by their task relevance [7–10]. For instance, when observers were presented with audiovisual signals with a small spatial disparity, they reported different spatial estimates depending on whether the visual or the auditory signal were task relevant [7]. Moreover, observers’ auditory spatial estimates were more variable than their visual estimates for collocated auditory and visual signals [7]. Recent Magnetoencephalography (MEG)/Electroencephalography (EEG) and functional magnetic resonance imaging (fMRI) studies have suggested that while posterior parietal cortices integrate signals weighted by their sensory reliabilities irrespective of task context, anterior parietal cortices encode spatial estimates depending on their reliability and task relevance [11–13]. In anterior parietal cortices, spatial estimates rely more on the location of the signals of the sensory modality that needs to be reported. While these behavioural and neuroimaging findings demonstrate that observers’ perceptual goals influence how the brain combines sensory signals to support perceptual inference, the underlying computational and neural mechanisms remain controversial [14,15].

**Fig 1:**
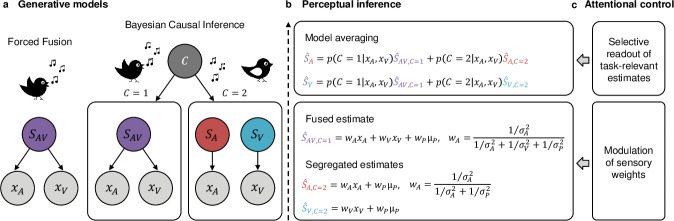
Bayesian Causal Inference and the possible roles of attentional control. **(a)** Generative models of Forced Fusion and Bayesian Causal Inference. For Forced Fusion, a single source generates auditory and visual signals. Bayesian Causal Inference explicitly models the two causal structures, i.e., whether auditory and visual signals come from one common cause (*C* = 1) or from separate causes (*C* = 2). **(b)** During perceptual inference, the observer is thought to invert the generative models; it infers the number of sources by combining prior knowledge and audiovisual evidence. A Forced Fusion estimate is computed by averaging auditory and visual estimates alone with prior spatial estimates weighted by their relative reliabilities (inverse sensory variance *σ*^2^). The full segregation estimates, visual or auditory, are computed separately. To account for causal uncertainty, the final Bayesian Causal Inference estimate, auditory (${\hat{S}}_{A}$) or visual (${\hat{S}}_{V}$), is computed by combining the audiovisual Forced Fusion estimate (${\hat{S}}_{AV,C=1}$) with the task-relevant full segregation estimate, auditory (${\hat{S}}_{A,C=2}$) or visual (${\hat{S}}_{V,C=2}$), each weighted by the posterior probabilities of a common (*C* = 1) or independent (*C* = 2) causes. **(c)** Attentional control can mould multisensory perceptual inference via two distinct mechanisms and thereby induce differences in observers’ auditory and visual estimates. First, attending to a particular sensory modality may enhance the reliability of the signals in the attended sensory modality and thereby their weights during Forced Fusion. Second, modality-specific report (i.e., task relevance) determines the late readout consistent with the principles of Bayesian Causal Inference, i.e., whether the Forced Fusion estimate is combined with the auditory or visual full segregation estimate.

Two mechanisms have been proposed. In the first “forced fusion account,” attention to one sensory modality, for instance, vision, enhances the precision (i.e., inverse of variance) of the visual representations [16–18] prior to fusion and thereby their weights in the sensory fusion process [8] (but see [19]). These task-dependent sensory weights naturally result in different perceptual estimates for auditory and visual report. In the second “causal inference account,” task-dependent perceptual estimates arise from observers’ causal uncertainty, i.e., their uncertainty about whether signals come from common or independent sources. Hierarchical Bayesian causal inference [20–26] accounts for this causal inference problem by explicitly modelling the possible underlying causal structures (Fig 1A and 1B). In the case of common sources, inputs are fused into one unified percept, weighted by their relative sensory reliabilities [1–6]. In the case of independent sources, signals are segregated. Critically, observers do not know the underlying causal structure and infer it from noisy spatial, temporal, and other higher-order statistical correspondences. To account for observers’ causal uncertainty, a final estimate of an environmental property (e.g., spatial location) is computed by combining the estimates under the assumptions of “common” and “independent” sources. According to a decisional strategy referred to as “model averaging” (for other decisional functions, see [27]), observers combine the forced fusion audiovisual estimate either with the auditory segregation estimate for auditory report or with the visual estimate for visual report weighted by the posterior probabilities of each respective causal structure. This late readout thus results in different perceptual estimates for auditory and visual report.

In short, two distinct mechanisms can lead to differences in neural representations and perceptual estimates for auditory and visual report conditions (Fig 1C). The first mechanism impacts sensory processing prior to and during sensory fusion, while the second mechanism relies on a late readout that flexibly combines the forced fusion and task-relevant segregation estimates. Crucially, all neuroimaging studies to date [7,11–13,28,29] have conflated these two distinct computational and neural mechanisms by instructing observers to report their percept in the same sensory modality throughout an entire run. As a result, differences between representations for auditory and visual report may have come from attentional modulation of sensory weights during stimulus processing and/or a late readout of the task-relevant estimate according to Bayesian causal inference.

The current study was designed to dissociate these two distinct mechanisms using a pre- and postcueing paradigm with a spatial localisation task. Participants were presented with synchronous audiovisual spatial signals at variable spatial disparities. A precue indicated the sensory modality that needed to be attended and a postcue whether the auditory or visual location needed to be reported. Combining psychophysics, fMRI and Bayesian modelling, we characterised the neural mechanisms by which the brain controls the combination of sensory signals depending on spatial disparity, prestimulus attention and poststimulus report along the auditory and visual dorsal spatial processing hierarchies. We expected prestimulus attention to alter the reliability of sensory representations in early sensory cortices and hence their weights in the initial sensory fusion process. By contrast, consistent with Bayesian causal inference, poststimulus report should mould how anterior parietal cortices flexibly combine sensory signals according to observers’ perceptual goals.

## Results

**Fig 2:**
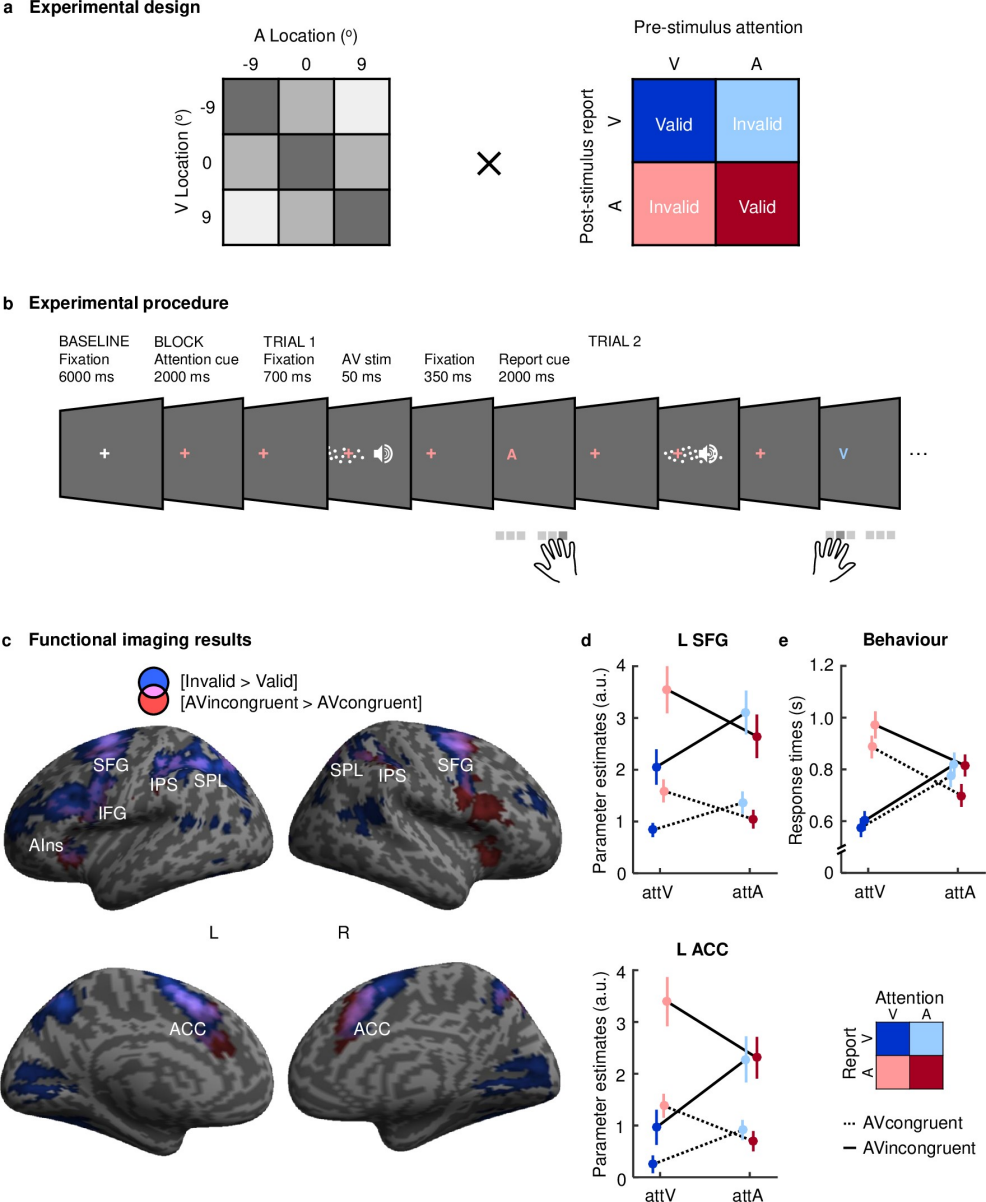
Experimental design and procedure, neuroimaging univariate results, and response times in the fMRI experiment. **(a)** The experiment conformed to a 3 (auditory location) × 3 (visual location) × 2 (prestimulus attention: attA, attV) × 2 (poststimulus report: repA, repV) factorial design (A for auditory and V for visual). Auditory and visual signals were independently sampled from 3 locations along the azimuth (−9°, 0°, and 9° visual angle), resulting in 9 audiovisual spatial combinations with 3 levels of spatial disparity: none (0°; dark grey); low (9°; mid grey); and high (18°; light grey). The orthogonal pre- and postcue attention cueing paradigm resulted in two valid (attArepA; attVrepV) and two invalid (attVrepA; attArepV) conditions. **(b)** Prior to block start, participants were cued to attend to either the auditory or visual signal (via colour of fixation cross); 350 ms after each audiovisual stimulus, they were cued to report their perceived auditory or visual location (via coloured letter: A for auditory and V for visual). Participants responded via a button press using different keypads for each sensory modality. **(c)** Increased activations for invalid relative to valid trials [Invalid (attVrepA & attArepV) > Valid (attArepA & attVrepV)] in blue, for AV spatially incongruent relative congruent stimuli [AVincongruent (AV disparity ≠ 0°) > AVcongruent (AV disparity = 0°)] in red and their overlap in pink, rendered on an inflated canonical brain (*p* < 0.001 uncorrected at peak level for visualisation purposes, extent threshold k > 0 voxels). **(d)** Across participants’ mean (±SEM) parameter estimates in arbitrary units from L SFG (x = −4, y = 8, and z = 52) and L ACC (x = −10, y = 18, and z = 32). **(e)** Across participants’ mean (±SEM) response times. Data in d and e plotted as a function of (i) prestimulus attention: auditory attA versus visual attV; (ii) poststimulus report: auditory repA versus visual repV; and (iii) audiovisual spatial (in)congruency: AVincongruent (AV disparity ≠ 0°) versus AVcongruent (AV disparity = 0°). The data used to make this figure are available in S1 and S2 Datas. ACC, anterior cingulate cortex; AIns, anterior insula; IFG, inferior frontal gyrus; IPS, intraparietal sulcus; L ACC, left anterior cingulate gyrus; L SFG, left superior temporal gyrus; SFG, superior temporal gyrus; SPL, superior parietal lobule.

In both a psychophysics experiment (outside the scanner) and a subsequent fMRI experiment, we presented observers with synchronous auditory and visual stimuli that were independently sampled from 3 locations along the azimuth, resulting in 3 levels of audiovisual spatial disparity (congruent: 0°; low disparity: 9°; and high disparity: 18° visual angle). Observers were precued to attend to either auditory or visual modalities prior to stimulus presentation and postcued to report either their perceived auditory or visual location (Fig 2A and 2B). In 50% of the trials, the precue was valid, i.e., observers attended to the sensory modality in which they had to report the spatial location poststimulus. In the other 50% of the trials, the precue was invalid, and observers had to switch attention between sensory modalities to report the spatial location of the postcued modality. This pre- and postcueing paradigm enabled us to dissociate how the brain controls multisensory perceptual inference via attention to one particular sensory modality prior or during stimulus processing (i.e., precue effect) and via flexible readout of the perceptual estimate in the task-relevant sensory modality poststimulus (i.e., postcue effect). In an additional unisensory localisation experiment inside the scanner, we confirmed that observers successfully located sounds despite the scanner noise and, as expected [30–33], showed greater activations for contralateral relative to ipsilateral sounds in superior temporal gyri, in particular plana temporalia (for details, see S1 Text, S1 Fig and S11 Table).

### Response times and voxel-wise BOLD responses in attentional control of multisensory processing

Using behavioural response times and voxel-wise BOLD responses, we first investigated how the brain controls audiovisual processing when the auditory and visual signals were spatially incongruent, i.e., presented at different locations. On these audiovisual conflict trials, observers need to suppress the influence of the interfering signal in the task-irrelevant sensory modality, typically leading to longer response times [34]. Consistent with this conjecture, observers were slower when responding to spatially conflicting than congruent signals (i.e., main effect of spatial disparity, psychophysics: *p* < 0.001, ηp^2^ = 0.72; fMRI: *p* < 0.001, ηp^2^ = 0.84). Further, this spatial incongruency effect was stronger for auditory than visual report, when the more reliable (i.e., visual) signal needed to be ignored [35]. In other words, we observed a significant interaction between spatial disparity and poststimulus report in response times for both the psychophysics (*p* < 0.001, ηp^2^ = 0.27) and fMRI (*p* < 0.001, ηp^2^ = 0.74) experiments (S1 and S2 Tables). At the neural level, locating a visual (or auditory) stimulus in the context of a spatially incongruent signal in another sensory modality increased activations in a frontoparietal insular system, previously implicated in conflict detection and attentional control [35,36]. Activation increases for spatially disparate relative to congruent stimuli were observed in the superior frontal gyri, the superior parietal lobules, the intraparietal sulci, the inferior frontal gyri, and the anterior insula (for comprehensive results, see S3 Table).

Next, we investigated how the brain responds when observers need to switch their attentional focus after the postcue, because the precue was invalid. Consistent with well-established attentional switching costs [37–41], observers were slower to respond when precues were invalid relative to valid (i.e., interaction between prestimulus attention and poststimulus report, psychophysics: *p* < 0.001, ηp^2^ = 0.90; fMRI: *p* < 0.001, ηp^2^ = 0.92; S1 and S2 Tables). This profile of response times suggests that observers allocated their attentional resources effectively between sensory modalities as instructed by the precues, even though the precues were valid only on 50% of the trials. Because observers used different response hands for the two different sensory modalities, interference at the response selection and motor processing level may also contribute to these switching costs.

At the neural level, invalid relative to valid trials increased activations in a bilateral frontoparietal system encompassing the superior frontal gyri, the intraparietal sulci, the precuneus, and the middle (and inferior) frontal gyri (S4 Table). This bilateral frontoparietal system was recruited irrespective of whether observers shifted their attention from the auditory to the visual sense or vice versa (S5 Table), which is consistent with the idea that intersensory reorienting relies on neural systems largely shared across the senses [42–45]. Our fMRI results thus further corroborate that observers shifted their attention from vision to audition and vice versa as indicated by the pre- and poststimulus cues.

Collectively, our results suggest that spatial conflicts between audiovisual signals and conflicts between pre- and postcues are associated with longer response times and activations in a widespread frontoparietal insular system previously implicated in cognitive control and selective attention [46] (Fig 2C–2E). Indeed, a formal so-called “conjunction null” conjunction analysis (i.e., a logical “AND”) revealed activation increases for invalid relative to valid trials and for audiovisual spatially incongruent relative to congruent trials in a partly overlapping bilateral frontoparietal insular system (Table 1).

**Table 1:** fMRI univariate results: Conjunction of cue invalidity and audiovisual spatial incongruency.

Brain regions	MNI coordinates (mm)			Cluster size (voxels)	z-score (peak)	p _FWE corrected_ (peak)
	**x**	**y**	**z**			
Invalid > Valid ∩ AVincongruent > AVcongruent						
R superior frontal gyrus	22	0	52	492	>8	0.000
L superior frontal gyrus	−24	−4	54	512	7.23	0.000
L anterior cingulate gyrus	−2	14	48	615	>8	0.000
R anterior cingulate gyrus	8	18	40		7.07	0.000
L anterior insula	−30	26	2	75	6.45	0.000
R superior parietal lobule	14	−68	54	96	6.14	0.000
L superior parietal lobule	−16	−70	52	70	5.73	0.000
R intraparietal sulcus	34	−44	46	25	5.14	0.006
L intraparietal sulcus	−34	−46	46	65	4.91	0.017

Conjunction null conjunction analysis to show common activations for [Invalid > Valid] ∩ [AVincongruent (AV disparity ≠ 0°) > AVcongruent (AV disparity = 0°)]. *p*-Values are FWE corrected at the peak level for multiple comparisons within the entire brain.

FWE, family-wise error; L, left; MNI, Montreal Neurological Institute; R, right.

### Computational mechanisms: Behavioural audiovisual weight index *w*_*AV*_ and Bayesian modelling

To investigate how the brain controls the weighting and combination of signals into spatial representations, we computed a behavioural audiovisual weight index *w*_*AV*_ separately for the 8 conditions in our 2 (prestimulus attention: auditory versus visual) × 2 (poststimulus report: auditory versus visual) × 2 (disparity: low versus high) design (S7A and S8A Tables). The behavioural audiovisual weight index *w*_*AV*_ quantifies the relative influence of the true auditory and visual signal locations on participants’ behavioural spatial reports for audiovisual spatial conflict trials. An audiovisual weight index *w*_*AV*_ of 1 indicates that the observer’s spatial report relies completely on the visual signal location. An audiovisual weight index *w*_*AV*_ of 0 indicates that the observer’s spatial report relies completely on the auditory signal location.

**Fig 3:**
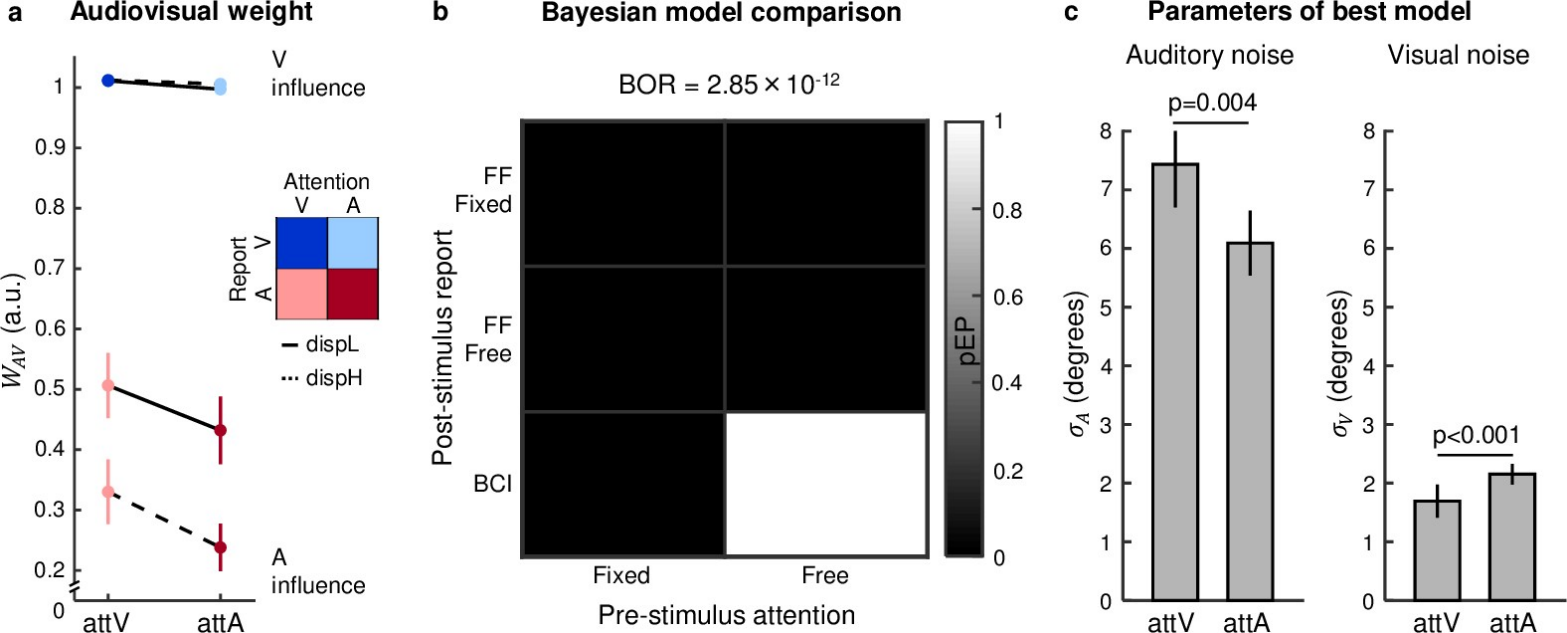
Audiovisual weight index (*w*_*AV*_) and Bayesian modelling results for the fMRI experiment. **(a)** Across participants’ mean *w*_*AV*_ (±SEM) shown as a function of (i) prestimulus attention: auditory attA versus visual attV; (ii) poststimulus report: auditory repA versus visual repV; and (iii) AV spatial disparity: low dispL (9°) versus high dispH (18°). *w*_*AV*_ = 1 for purely visual and *w*_*AV*_ = 0 for purely auditory influence. **(b)** Along the first factor of a 2 × 3 factorial model space, we assessed the influence of prestimulus attention by comparing whether the sensory variances were (i) constant (fixed: $\sigma_{A\;attA}^{2}$ = $\sigma_{A\;attV}^{2}$, $\sigma_{V\;attA}^{2}$ = $\sigma_{V\;attV}^{2}$); or (ii) different (free: $\sigma_{A\;attA}^{2}$, $\sigma_{A\;attV}^{2}$, $\sigma_{V\;attA}^{2}$, $\sigma_{V\;attV}^{2}$) across prestimulus attention. Along the second factor, we assessed the influence of poststimulus report by comparing (i) a forced fusion model in which the sensory variances were fixed (FF fixed: $\sigma_{A\;repA}^{2}$ = $\sigma_{A\;repV}^{2}$, $\sigma_{V\;repA}^{2}$ = $\sigma_{V\;repV}^{2}$); (ii) a forced fusion model in which the sensory variances were allowed to differ between auditory and visual report (FF free: $\sigma_{A\;repA}^{2}$, $\sigma_{A\;repV}^{2}$, $\sigma_{V\;repA}^{2}$, $\sigma_{V\;repV}^{2}$); and (iii) a BCI model in which the influence of poststimulus report arises via a late flexible readout. The matrix represents our 2 × 3 model space. For each model, we show the pEP (larger pEP represents better model) via greyscale. BOR represents the probability that results are due to chance. **(c)** Across participants’ mean (±SEM) of auditory and visual noise parameter estimates (i.e., $\sigma_{A\;attA}^{2}$, $\sigma_{A\;attV}^{2}$, $\sigma_{V\;attA}^{2}$, $\sigma_{V\;attV}^{2}$) of the best model, i.e., BCI model with free prestimulus attention parameters (attA, auditory; attV, visual). *p*-Values based on one-tailed sign permutation test. The data used to make this figure are available in S2 Data. BCI, Bayesian causal inference; BOR, Bayesian omnibus risk; FF, Forced Fusion; pEP, protected exceedance probability.

As shown in Fig 3A, the behavioural audiovisual weight index *w*_*AV*_ depended on both prestimulus attention and poststimulus report. First, we observed a significant main effect of prestimulus attention (psychophysics: *p* = 0.007, es = 0.05 [0.02, 0.07]; fMRI: *p* = 0.002, es = 0.05 [0.01, 0.08], one-tailed permutation test, es = effect size [95% CI]). The *w*_*AV*_ index shifted towards 1 (i.e., into the direction of pure visual influence) for attention to vision and towards 0 for attention to audition, suggesting that the focus of prestimulus attention influenced how the brain weights audiovisual signals for perceptual inference. Second, we observed a significant main effect of poststimulus report (psychophysics: *p* < 0.001, es = 0.54 [0.48, 0.61]; fMRI: *p* < 0.001, es = 0.63 [0.54, 0.73], one-tailed permutation test, es = effect size [95% CI]). Contrary to the predictions of forced fusion models [1–6], observers did not integrate sensory signals weighted by their sensory reliabilities into one unified percept, hence reporting the same location for the auditory and visual stimulus. Instead, the influence of the auditory and visual signals on observers’ percept depended on their task relevance. Observers relied more on the auditory signal when reporting the auditory location and more on the visual signal when reporting the visual location. Critically, the difference in *w*_*AV*_ between auditory and visual report also depended on spatial disparity: This difference was smaller for low than high AV spatial disparity trials, when signals are less likely to originate from a common source [20] (psychophysics: *p* < 0.001, es = 0.09 [0.07, 0.11]; fMRI: *p* < 0.001, es = 0.09 [0.07, 0.11], es = effect size [95% CI]). This profile was observed in particular for the auditory report conditions (i.e., significant poststimulus report × AV disparity interaction, psychophysics: *p* < 0.001, es = 0.19 [0.16, 0.23]; fMRI: *p* < 0.001, es = 0.19 [0.16, 0.22], es = effect size [95% CI]), where the influence of the visual location on *w*_*AV*_ declined with higher AV spatial disparities (psychophysics: *p* < 0.001, es = 0.19 [0.15, 0.22]; fMRI: *p* < 0.001, es = 0.18 [0.15, 0.22], es = effect size [95% CI]).

Collectively, our results show that both prestimulus attention and poststimulus report influence how observers weight and combine signals to support perceptual inference. Both visual prestimulus attention and poststimulus report shifted the behavioural *w*_*AV*_ index towards 1, i.e., towards stronger visual influence. Yet, only the effects of poststimulus report but not of prestimulus attention depended on spatial disparity raising the question whether the two effects rely on different computational mechanisms [11,12,47]. Prestimulus attention may affect the audiovisual weight index by reducing the sensory noise or variance of the attended sensory signals and thereby their weights in the fusion process. By contrast, poststimulus report may affect the audiovisual weight index by later determining a perceptual readout that flexibly combines that audiovisual fusion with the full segregation estimate of the to-be-reported sensory modality.

To formally investigate whether the effects of prestimulus attention and poststimulus report are mediated by different computational mechanisms, we compared 6 Bayesian models organised in a 2 (prestimulus) × 3 (poststimulus) factorial model space (Fig 3B). Along factor 1, we assessed the influence of prestimulus attention: we manipulated whether the auditory and visual variances were (i) fixed or (ii) allowed to differ between auditory and visual prestimulus attention (i.e., auditory variance: $\sigma_{A\;attA}^{2}$, $\sigma_{A\;attV}^{2}$; visual variance: $\sigma_{V\;attA}^{2}$, $\sigma_{V\;attV}^{2}$). Along factor 2, we assessed the impact of poststimulus report on observers’ percept. We compared (i) a forced fusion model in which the sensory variances were fixed across poststimulus report; (ii) a forced fusion model in which the sensory variances were allowed to differ between auditory and visual poststimulus report (i.e., auditory variance: $\sigma_{A\;repA}^{2}$, $\sigma_{A\;repV}^{2}$; visual variance: $\sigma_{V\;repA}^{2}$, $\sigma_{V\;repV}^{2}$); and (iii) a Bayesian causal inference model in which the influence of poststimulus report arose via a late readout that flexibly combines the forced fusion with the full segregation estimates. Bayesian model comparison of the 6 models in our 2 × 3 model space provided overwhelming evidence for the Bayesian causal inference model that includes a modulatory effect of prestimulus attention on the sensory variances (protected exceedance probability ≈ 1; Bayesian Omnibus Risk = 2.85 × 10^−12^). This result suggests that observers control multisensory inference via two distinct mechanisms (for comprehensive results, see S10 Table). First, modality-specific prestimulus attention increases the reliability of the attended sensory inputs prior to and during sensory fusion. Second, modality-specific poststimulus report determines a late readout that flexibly combines a forced fusion estimate with the unisensory estimate in the task-relevant modality consistent with Bayesian causal inference. Our results from formal Bayesian model comparison dovetail nicely with our analysis of the audiovisual weight index *w*_*AV*_ showing that the effect of poststimulus report (but not prestimulus attention) depend on spatial disparity [11–13]. This interaction between poststimulus report and spatial disparity can be explained by Bayesian causal inference [20,21]. When signals are far apart in space and hence likely to come from separate sources, the influence of the task-relevant full segregation estimate is stronger on observers’ final estimate, resulting in a greater difference in *w*_*AV*_ between auditory and visual poststimulus report.

Focusing on the winning model, we compared the auditory and visual variances across the two prestimulus attention conditions (Fig 3C). This analysis confirmed that the auditory variance $\sigma_{A}^{2}$ significantly decreased for auditory relative to visual attention (psychophysics: *p* = 0.009, es = 2.33 [1.29, 3.38]; fMRI: *p* = 0.004, es = 1.34 [0.34, 2.35], one-tailed permutation test, es = effect size [95% CI]), while the visual variance $\sigma_{V}^{2}$ significantly decreased for visual relative to auditory attention (psychophysics: *p* = 0.014, es = 0.34 [0.06, 0.62]; fMRI: *p* < 0.001, es = 0.46 [0.04, 0.88], one-tailed permutation test, es = effect size [95% CI]).

### Neural mechanisms: Multivariate decoding and neural audiovisual weight index *nw*_*AV*_

**Fig 4:**
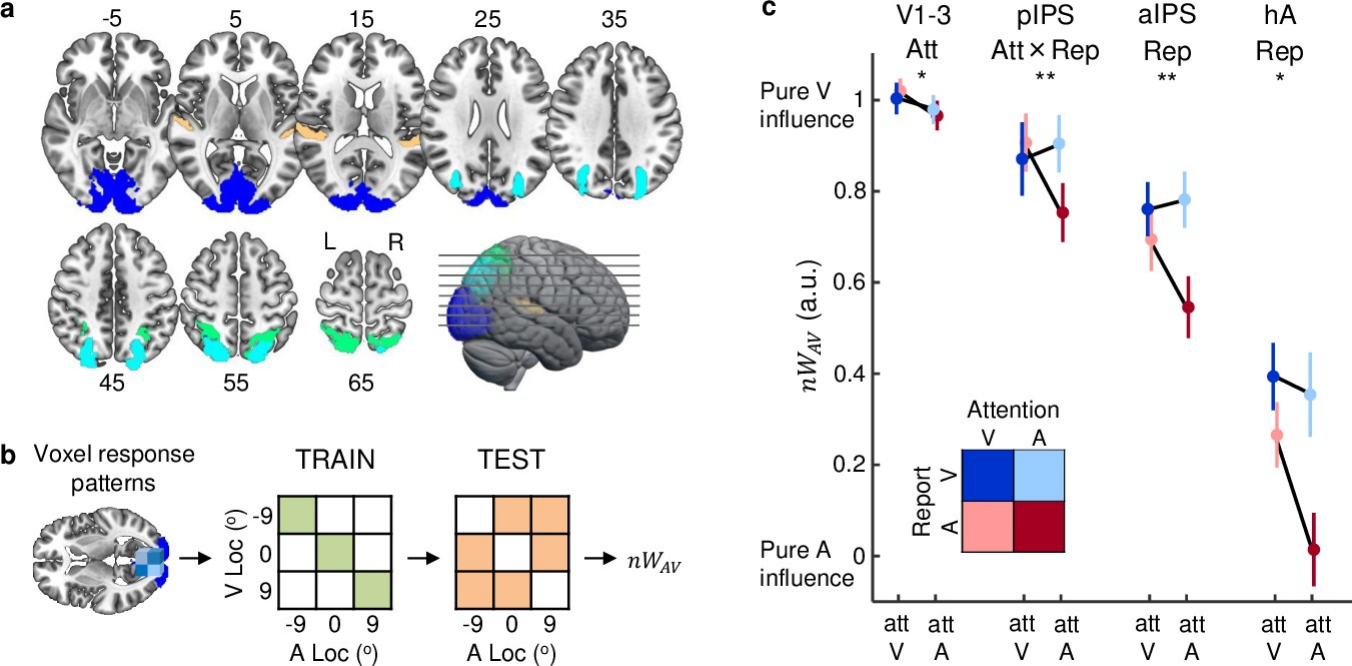
Neural audiovisual weight index (*nw*_*AV*_) across the audiovisual processing hierarchy. **(a)** fMRI voxel response patterns were obtained from anatomical ROIs along the visual and auditory dorsal cortical hierarchies: V1-3 (blue), pIPS (cyan), aIPS (green), and hA (orange). ROIs are displayed on a canonical brain. **(b)** An SVR model was trained to learn the mapping from the fMRI voxel response patterns to the external spatial locations based on the audiovisual spatially congruent trials (green cells = congruent). The learnt mapping was then used to decode the spatial location from the fMRI voxel response patterns of the audiovisual spatially incongruent trials (orange cells = incongruent) to compute *nw*_*AV*_. **(c)** Across participants’ mean *nw*_*AV*_ (±SEM) shown as a function of (i) prestimulus attention (Att): auditory/attA versus visual/attV; and (ii) poststimulus report (Rep): auditory/repA versus visual/repV, with statistical results of sign permutation tests. *nw*_*AV*_ = 1 for purely visual and *nw*_*AV*_ = 0 for purely auditory influence. The data used to make this figure are available in S2 Data. ** *p* < 0.01, * *p* < 0.05. aIPS, anterior intraparietal sulcus; hA, higher-order auditory cortex; pIPS, posterior intraparietal sulcus; ROI, region of interest; SVR, support vector regression; V1-3, low-level visual cortex.

Combining fMRI and multivariate pattern decoding, we next investigated how the brain combines auditory and visual signals depending on prestimulus and poststimulus cues into spatial representations along the dorsal visual and auditory processing hierarchies (Fig 4A). We trained a linear support vector regression (SVR) model to learn the mapping from BOLD response patterns to external physical stimulus location based on audiovisual spatially congruent trials (for details, see [11,12]). We first confirmed that all regions of interest (ROIs) reliably encoded the spatial locations of audiovisually congruent stimuli. Indeed, the Pearson correlation coefficients between the decoded locations and the true physical locations of audiovisual congruent trials were significantly greater than 0 [95% CI] in low-level visual cortex (V1-3: 0.88 [0.83, 0.91], *p* < 0.001, es = 1.36 [1.19, 1.52]), posterior intraparietal sulcus (pIPS: 0.56 [0.40, 0.69], *p* < 0.001, es = 0.63 [0.42, 0.84]), anterior intraparietal sulcus (aIPS: 0.39 [0.32, 0.46], *p* < 0.001, es = 0.41 [0.33, 0.49]), higher-order auditory cortex (hA: 0.16 [0.07, 0.25], *p* = 0.003, es = 0.16 [0.07, 0.25]), and low-level auditory cortex (A: 0.08 [0.04, 0.13], *p* = 0.003, es = 0.08 [0.03, 0.13]).

We next used this mapping (i.e., learnt from audiovisual congruent trials) to decode the spatial locations from BOLD response patterns elicited by audiovisual incongruent stimuli (Fig 4B; for details, see [11,12]). Consistent with the analysis of behavioural localisation responses, we computed a neural audiovisual weight index *nw*_*AV*_ that quantifies the relative influence of the true visual and auditory locations on the decoded locations of audiovisual incongruent stimuli. As shown in Fig 4C, the neural audiovisual weight index *nw*_*AV*_ was not statistically different from 1 in early visual cortices (V1-3: mean *nw*_*AV*_ = 0.99, *p* = 0.478, es = −0.01 [−0.07, 0.05], one-tailed permutation test on *nw*_*AV*_ < 1), indicating almost purely visual influence on spatial representations. In posterior and anterior parietal cortices, the *nw*_*AV*_ shifted overall towards lower values (pIPS: mean *nw*_*AV*_ = 0.86, *p* = 0.292, es = 0.14 [0.03, 0.25]; aIPS: mean *nw*_*AV*_ = 0.69, *p* = 0.061, es = 0.31 [0.23, 0.40], one-tailed permutation test on *nw*_*AV*_ < 1), consistent with the notion that multisensory interactions increase progressively along the cortical hierarchy [36,48–58]. In auditory cortices, the *nw*_*AV*_ index was closer to 0, but still significantly different from 0 (A: mean *nw*_*AV*_ = 0.25, *p* = 0.024, es = 0.25 [0.03, 0.47]; hA: mean *nw*_*AV*_ = 0.24, *p* = 0.004, es = 0.24 [0.10, 0.37], one-tailed permutation test on *nw*_*AV*_ > 0), indicating considerable visual influence on spatial representations. These substantial visual influences on spatial representations in auditory cortices reflect the greater spatial reliability of the visual inputs in our study, in line with our current and past behavioural results [11–13,47]. Moreover, while visual location is encoded retinotopically in visual cortices, in posterior auditory cortices sound location is encoded in the relative activity of two neuronal populations, broadly tuned to ipsi- or contralateral hemifields (i.e., “hemifield code”) [59,60] making the fMRI decoding of the sound location potentially less reliable and thereby more susceptible to visual influences. More specifically, two main components contribute to sound location encoding in auditory cortices. First, auditory cortices show a response bias to the contralateral hemifield, such that the relative activations across the left and right auditory cortices are informative about the sound location along the azimuth. Second, even though auditory cortices lack a topographic organisation for space, previous neuroimaging research [59] has shown that voxels differ in their azimuthal “tuning functions.” In particular, voxels in anterior and posterior regions of auditory cortices exhibited a more pronounced negative BOLD response to ipsilateral stimuli. Hence, the fine-grained activation pattern even within either left or right auditory cortices may be informative about sound location. To further dissociate these two contributions to sound location encoding, we also assessed decoding accuracy for SVMs that were trained separately on BOLD response patterns of left and right higher-order auditory cortices. The Pearson correlation coefficients between the decoded locations and the true physical locations of audiovisual congruent trials were significantly greater than 0 [95% CI] in left hA (0.08 [0.01, 0.16], *p* = 0.016, es = 0.08 [0.01, 0.16]) but not in right higher-order auditory cortex (right hA: −0.08 [−0.14, −0.01], *p* = 0.972, es = 0.08 [0.01, 0.15]). Consistent with [59,60], these supplementary results suggest that fMRI decoding of sound location from auditory cortices relies on two mechanisms, the relative BOLD responses between left and right auditory cortices as well as a more patchy functional organisation within the auditory cortex of each hemisphere.

Next, we used the audiovisual weight index *nw*_*AV*_ to investigate how the audiovisual signals are weighted and combined into spatial representations along the visual and auditory processing hierarchies depending on prestimulus attention and poststimulus report. Our results (S7B and S8B Tables) show a double dissociation of the effects of prestimulus attention and poststimulus report on the neural audiovisual weight indices. Prestimulus attention influenced the sensory weights in early visual cortices (i.e., main effect in V1-3: *p* = 0.011, es = 0.03 [0.01, 0.06], one-tailed permutation test). As expected, the visual influence on sensory weights was enhanced under visual prestimulus attention. By contrast, poststimulus report influenced the sensory weights in anterior parietal cortices (aIPS: *p* = 0.002, es = 0.15 [0.07, 0.23], one-tailed permutation test) and in planum temporale (hA, *p* = 0.030, es = 0.17 [0.01, 0.34], one-tailed permutation test). Again as expected, the *nw*_*AV*_ index was greater for visual than auditory poststimulus report. Finally, we observed a significant prestimulus attention × poststimulus report interaction in posterior parietal cortices (pIPS: *p* = 0.006, es = 0.17 [0.07, 0.28]). Post hoc one-tailed permutation testing revealed that the *nw*_*AV*_ index was greater (i.e., shifted towards visual influence) for visual than auditory prestimulus attention under auditory poststimulus report (*p* = 0.007, es = 0.14 [0.04, 0.23]). Thus, in line with our predictions, influences of prestimulus attention were revealed when the spatially less reliable auditory signal needs to be reported. Collectively, our decoding results show that prestimulus attention and poststimulus report influence the weighting and combination of audiovisual signals into spatial representations at distinct levels of the cortical hierarchy.

## Discussion

Combining psychophysics, fMRI multivariate decoding and Bayesian modelling, we investigated how observers control the combination of sensory signals into spatial representations guided by goals and task demands. In an attentional pre- and postcueing paradigm, we precued participants to attend to audition (or vision) and postcued them to report their perceived auditory (or visual) location. Our results show that the brain controls multisensory inference via two distinct neural mechanisms at different levels of the cortical hierarchy.

In our behavioural analysis, observers were slower when they had to switch attention across the senses on trials with invalid precues. These attentional switching costs suggested that observers shifted their attention effectively as instructed by the pre- and postcues [37–41]. At the neural level, intersensory reorienting relied on a widespread frontoparietal insular system previously implicated in mechanisms of conflict detection, selective attention, and cognitive control [42–46]—further corroborating the effectiveness of our attentional manipulation. Longer response times and activations in this frontoparietal insular system also arose for spatially disparate audiovisual trials that required observers to eliminate incongruent information from the unreported modality. Collectively, these behavioural and neural results show that observers control multisensory processing via a widespread frontoparietal insular system particularly on trials that involve reorienting between the senses or processing of incongruent sensory information. Our results converge with previous research showing a pivotal role for prefrontal cortices in arbitrating between sensory integration and segregation [61]. More specifically, prefrontal cortices were shown to control multisensory processing by combining prior information with cross-sensory correspondences such as temporal, spatial, or higher-order statistical congruency cues (e.g., phonetics) [35,62].

Next, we investigated how the brain controls the combination of conflicting audiovisual signals into spatial representations depending on prestimulus attention and poststimulus report. We characterised the influence of auditory and visual signals on observers’ percept with an audiovisual weight index *w*_*AV*_ at the behavioural and neural level. Our behavioural results show that both prestimulus attention to vision and visual poststimulus report increase the influence of the visual location on observers’ reported percept. Critically, only the effect of poststimulus report—but not of prestimulus attention—was greater for high relative to low audiovisual spatial disparity, raising the possibility that the two effects rely on different mechanisms [11,12,47]. Consistent with this conjecture, Bayesian modelling and model comparison showed that the behavioural data were best explained by a model in which prestimulus attention and poststimulus report influenced audiovisual processing via different computational mechanisms. In the winning model, the effect of prestimulus attention was mediated by changes in auditory and visual reliabilities. This converges with previous research showing greater sensory variances when observers divided their attention across sensory modalities than when they focused their attention on one particular sensory modality prior to stimulus presentation [10]. By contrast, the effects of poststimulus report were accommodated by the structure of the Bayesian causal inference model, which forms a final percept by flexibly combining the spatial estimates formed under the assumptions of common and independent sources according to various decision functions (e.g., model averaging [27]). For instance, when the visual location needs to be reported, the forced fusion audiovisual estimate is averaged with the full segregation visual estimate, each weighted by the posterior probabilities of the respective causal structure. Because the weight of the full segregation estimate is stronger when it is unlikely that the two signals come from a common source, Bayesian causal inference also provides a principled explanation for the fact that the difference between auditory and visual reports is greater at large spatial disparities. In summary, both the audiovisual weight index *w*_*AV*_ and formal Bayesian model comparison show that prestimulus attention and poststimulus report mould multisensory processing via different computational mechanisms. Prestimulus attention to vision increases the precision of the visual inputs and thereby their weights in sensory fusion. Poststimulus report relies on a late flexible readout of the task-relevant estimate consistent with Bayesian causal inference. These profound effects of modality-specific attention on integration of signals into spatial representations converge with previous research comparing modality-specific and divided attention [10]. Yet, they may challenge conclusions from early seminal behavioural studies in which spatial ventriloquism proved immune to modulatory effects of endogenous [63] and exogenous [64] spatial attention. It may be more difficult to reveal an effect of spatial attention, because it can exert counteracting effects on the emergence of the ventriloquist illusion by increasing the spatial precision of the attended sensory input as well as observers’ causal prior (i.e., their tendency to bind audiovisual signals within the spatial attentional spotlight; for further discussion, see [15]).

Combining fMRI and multivariate pattern decoding, we next investigated how the brain combines audiovisual signals into spatial representations. We first confirmed that ROIs along the auditory and visual dorsal processing hierarchies reliably encode spatial information as indicated by the significant correlation between the fMRI decoded locations and the true locations of audiovisual congruent stimuli. Next, we computed the neural audiovisual weight index *nw*_*AV*_ from the audiovisual incongruent trials to assess how the brain weights auditory and visual signals along the auditory and visual processing streams. As expected, the spatial representations in early visual and posterior parietal cortices were dominated by the location of the visual stimulus, while those in low-level auditory cortices and planum temporale reflected more the location of the auditory stimulus. Moreover, while we observed significant influences of visual signal location on spatial representations in low-level auditory cortices (i.e., significant: *nw*_*AV*_ > 0), these crossmodal influences were modest compared to those observed in posterior and parietal cortices. These findings converge with accumulating evidence showing multisensory interactions ubiquitously in human neocortex. Yet, even though multisensory interactions start already at the primary cortical level, they increase progressively across the cortical hierarchy [36,48–58].

Most importantly, we used the neural audiovisual weight index *nw*_*AV*_ to assess the influence of prestimulus attention and poststimulus report on how the brain combines auditory and visual signals into spatial representations at the neural level. We observed a double dissociation for the effects of prestimulus attention and poststimulus report along the cortical hierarchy. In early visual cortices, only prestimulus attention to vision increased the neural audiovisual weight index *nw*_*AV*_. In posterior parietal cortices, we observed a significant interaction between prestimulus attention and poststimulus report. Here, prestimulus attention to vision increased the visual influence on spatial representations mainly during auditory report, while no significant effects of prestimulus attention were observed for visual report. Finally, in anterior parietal cortices and planum temporale, we selectively observed a main effect of poststimulus report: when the visual location was task relevant and needed to be reported, the audiovisual weight index increased, indicating a stronger visual influence on spatial coding in anterior parietal cortices.

Collectively, our computational and neural results demonstrate that observers control audiovisual processing via two distinct mechanisms that act on different levels of the cortical hierarchy. Attention prior to and during stimulus processing increases the reliability of the attended (here: visual) signals and thereby their weight in the sensory fusion process in early visual and posterior parietal cortices, which is in line with the wealth of research showing early attentional effects at the primary cortical and even thalamic level [16–18].

Conversely, the influence of poststimulus report on spatial representations in parietal cortices converges with recent fMRI/EEG research, suggesting that Bayesian causal inference is performed by the brain via the dynamic computation of multiple spatial estimates across the cortical hierarchy [7,11–13]. Only at the top of the hierarchy, at about 350 to 450 ms, did anterior parietal areas combine auditory and visual signals into spatial estimates depending on their task relevance, i.e., whether the auditory or the visual location needed to be reported. Crucially, however, even though this past research attributed the differences in spatial representations (and neural weight indices) in anterior parietal cortices to mechanisms of Bayesian causal inference, these studies were not able to control for concurrent attentional effects on sensory reliability and weights [1–6], which the current study has shown to occur during stimulus processing. This is because past paradigms made observers report either their auditory or their visual percept throughout the entire run and thereby conflated effects of prestimulus attention and poststimulus report. The effect of task relevance could therefore have arisen either by increasing the reliability of the attended signal during stimulus processing or via a late selective readout of the task-relevant estimate according to Bayesian causal inference. The current study is therefore critical to corroborate the role of anterior parietal cortices in Bayesian causal inference. Intriguingly, because the poststimulus cue appeared 350 ms after stimulus offset, our current results suggest that the brain computes fusion and segregation estimates automatically during stimulus processing, it encodes and maintains these component estimates in working memory, and only 350 ms after stimulus offset it combines them flexibly into final perceptual estimates according to postcue instructions consistent with Bayesian causal inference. This late flexible readout may rely on heterogeneous populations of neurons that combine sensory signals with different weights as recently shown for the lateral intraparietal area [65] in neurophysiology of nonhuman primates. Future research exploiting the high temporal resolution of M/EEG or neurophysiology is needed to temporally resolve the influences of prestimulus attention and poststimulus report on multisensory integration and segregation across the cortical hierarchy. For instance, previous research has shown differences in early multisensory interactions at about 50 ms poststimulus for divided relative to modality-specific prestimulus attention [66], which, at a computational level, may reflect changes in sensory variances or the influence of an altered binding prior [10].

To conclude, the present study demonstrates that the brain controls how the brain weights and combines sensory signals for perceptual inference via two distinct computational and neural mechanisms that arise at distinct levels of the cortical hierarchy. Prestimulus attention enhances the precision of the attended inputs and thereby their weights during audiovisual fusion. Thus, attended visual inputs gain a stronger impact on spatial representations in early visual and posterior parietal cortices. Poststimulus report moulds how anterior parietal cortices read out representations that flexibly combine component estimates under the assumptions of common and independent sources, consistent with Bayesian causal inference.

## Methods

### Participants

The study included an initial psychophysics experiment outside the scanner followed by an fMRI experiment. A total of 35 volunteers participated in the initial psychophysics experiment. Moreover, 8 of those volunteers were excluded based on a priori exclusion criteria (see section “Exclusion and inclusion criteria”). As a result, 27 participants (10 males; mean age 20.5, range 18 to 30 years) were included in the analysis and results of the psychophysics experiment outside the scanner. This final number of included participants was based on a priori power analysis (G*Power 3.1[67]), with power (1-β) = 0.8, α = 0.05 and effect size Cohen’s d_AV_ = 0.5. Estimation of effect size for the psychophysics study was derived from the main effect of prestimulus attention on *w*_*AV*_ in a preliminary pilot study.

A total of 12 participants of the psychophysics experiment took part in the subsequent fMRI study. The fMRI sample size was determined based on previous neuroimaging experiments that used similar experimental designs, highly reliable estimation within each participant (i.e., 4 days of fMRI data acquisition per participant), and similar analysis approaches [11–13]. Participants included in the fMRI study (5 males; mean age 21.67 years, range 18 to 30 years) were right-handed according to the Edinburgh Handedness Inventory [68] (mean laterality index: 88.64; range: 60 to 100). We selected the first 12 participants from the initial psychophysics experiment that fulfilled the inclusion criteria for the fMRI experiment and stopped fMRI data acquisition after 12 participants were included. We did not post hoc exclude participants of the fMRI experiment (i.e., all fMRI datasets together with the associated behavioural responses inside the scanner were included in the final analysis). All volunteers reported normal or corrected to normal vision, normal hearing, and no history of neurological or psychiatric conditions.

### Ethics statement

All volunteers provided written informed consent and received financial reimbursement or course credits for their participation in the experiment. The study was approved by the ethical review committee of the University of Birmingham (approval number: ERN_11_0470AP4) and was conducted in accordance with the principles outlined in the Declaration of Helsinki.

### Exclusion and inclusion criteria—Psychophysics experiment

Volunteers were excluded post hoc from the psychophysics experiment based on two criteria. First, in a unisensory auditory or visual localisation screening observers located either auditory or visual signals that were randomly presented at −9°, 0°, or 9° visual angle along the azimuth. Their auditory and visual localisation accuracy was quantified by the root mean square error (RMSE) between participants’ reported location and signal’s true location. Observers were excluded as outliers if their RMSE was greater than 5.5° for auditory localisation or 3.5° for visual localisation (thresholds defined as two standards deviations above the group mean in a preliminary pilot study). A total of 4 volunteers were excluded based on RMSE greater than 5.5° for auditory localisation. Second, we expected observers to be significantly slower on trials when the prestimulus cue was invalid rather than valid (i.e., so-called switching costs [37–41]). By assessing these attentional switching costs, the second criterion ensures that we excluded observers that did not shift their attention as instructed by the precue. Specifically, observers were excluded if they did not show a significant cue validity effect (i.e., interaction between prestimulus attention and poststimulus report at *p* < 0.05) for response times in the pre- and post-cuing paradigm (see section “Experimental design”). Based on this criterion, 4 volunteers were excluded. This second criterion is very important, because the precue for shifting attention to the auditory or visual modality was valid only in 50% of the trials (in order to avoid additional effects of expectation and maximise design efficiency). In other words, we had to rely on observers that conscientiously followed task instructions even though shifting attention was not beneficial for task performance in our study, because the precue was uninformative.

### Exclusion and inclusion criteria—fMRI experiment

Participants of the psychophysics study were eligible for the subsequent fMRI experiment if they maintained central fixation throughout each run. We defined saccades as eye movements outside 1.3° circular area centred on participant’s median of fixation based on calibration trials [69]. Only participants who produced less than 20 saccades per run (i.e., 216 trials; threshold defined as two standards deviations above the group mean in a preliminary pilot study) were considered eligible to the fMRI experiment, until we reached a predefined sample size (see section “Participants”).

### Stimuli

The auditory stimulus was a burst of white noise (96,000 Hz sampling frequency; 65-dB sound pressure level; 50-ms duration, 5-ms on/off ramp) convolved with spatially selective head-related transfer functions (HRTFs) based on the KEMAR dummy head of the MIT Media Laboratory [70]. HRTFs from the locations in the database were interpolated to obtain the locations required for the study. The visual stimulus was a cloud of 20 white dots (luminance: 169 cd/m^2^; dot diameter: 0.3° visual angle, 50-ms duration) sampled from a bivariate Gaussian distribution with a vertical standard deviation of 1° visual angle and a horizontal standard deviation of 5° visual angle presented on a grey background (17 cd/m^2^). Guided by [47], we opted for relatively high visual reliability to ensure that observers can perform causal inference and arbitrate successfully between sensory integration versus segregation. Yet, high reliability of the physical visual inputs may reduce our ability to reveal subtle attentional effects on observers’ internal visual uncertainty. In short, our experiment was optimised to assess observers’ causal inference and attentional effects on observers’ reported sound percept.

The white noise bursts and cloud of white dots were generated independently for each experimental trial to prevent observers from using stimulus-specific cues for spatial localisation.

### Experimental design

The psychophysics experiment outside the scanner was followed by the fMRI experiment on separate days. Both experiments used identical designs that combined spatial ventriloquism with an attentional pre- and postcuing paradigm (Fig 2A). Observers were precued to attend to the auditory (or visual) modality (i.e., modality-specific prestimulus attention: attA or attV) at the beginning of each block. Next, they were presented with synchronous auditory and visual signals that were sampled independently from 3 positions along the azimuth (−9°, 0°, or 9° visual angle), leading to 3 levels of audiovisual spatial disparity (0°, 9°, and 18° visual angle). After stimulus presentation, they were postcued to report their perceived location of either the auditory or visual signal (i.e., modality-specific poststimulus report: repA or repV) using different keypads for each sensory modality. On 50% of the trials, the precue was valid: observers were precued to attend to the sensory modality (e.g., visual), and they were postcued to report (e.g., visual) after stimulus presentation. On the other 50% of the trials, the precue was invalid: observers were precued to attend to one sensory modality and postcued to report the location of the stimulus of the other sensory modality. In summary, the experiment conformed to a 2 (prestimulus attention: auditory versus visual via precue) × 2 (poststimulus report: auditory versus visual via postcue) × 3 (visual location) × 3 (auditory location) factorial design.

We deliberately made the precue noninformative for two reasons. First, such a balanced design is most efficient (i.e., we obtain the most reliable parameter estimates across all conditions). Second, we ensured that modality-specific prestimulus attention was not confounded by modality-specific expectation. This is important because accumulating research has shown that expectation and attention may rely on partly distinct neural mechanisms [71–74]. Importantly, an uninformative attentional precue (i.e., only valid in 50% of the trials) relies selectively on humans’ ability to shift their attention voluntarily, even if it does not incur any benefits for task performance. We post hoc ensured that the data included in the analysis (see section “Participants”) were from observers that shifted their attention as instructed by the precue, as indicated by switching costs on trials where the precue was invalid (see section “Exclusion and inclusion criteria—Psychophysics experiment”). Yet, it is well established that attention and expectation are intimately related. It is rational to direct attention to spatial locations or modalities when one expects potentially relevant events to happen. We would therefore expect that the effects of prestimulus attention may be stronger for informative cues (e.g., 75% validity).

Modality-specific prestimulus attention was manipulated over blocks of 12 trials, while prestimulus cue validity varied pseudo-randomly across trials to avoid effects of response expectations. At the beginning of each prestimulus attention block (Fig 2B), participants were instructed by a 2-second precue (i.e., colour of the fixation cross) to attend to either the auditory or the visual modality. Each trial within a prestimulus attention block started with 700-ms presentation of a coloured fixation cross indicating the attended sensory modality. Next, audiovisual spatial signals of variable locations and spatial disparity were presented in synchrony for 50 ms. After a 350-ms fixation interval, a postcue (i.e., coloured letter) indicated whether observers needed to report the location of the visual or auditory signal (i.e., “A” to locate the auditory signal; “V” to locate the visual signal) within a 2-second time interval. Both pre- and poststimulus cues were visual only to minimise the cue duration and hold the cue constant across all conditions. We acknowledge that this experimental choice may have given visual signals a processing benefit. However, because this potential processing benefit was constant across all conditions, it does not confound our effects of prestimulus attention and poststimulus report.

Importantly, observers indicated their perceived visual or auditory location via a three-button key press using different hands and keypads for the auditory and visual modality. We excluded all trials on which observers first responded with the wrong keypads. This ensures that cross-modal biases cannot be attributed to observers being confused about which sensory modality is task relevant and should be reported.

Prior to the main experiment, observers were trained in brief unisensory practice runs (10 trials per location and sensory modality) to learn the mapping from spatial locations to button responses. After each response, the participants received visual feedback on their response accuracy: the fixation cross turned green (respectively red) after a correct (respectively incorrect) response.

Every two prestimulus attention blocks, we presented a 6-second fixation block, which were indicated by a change in the colour of the fixation cross. Each run comprised 18 prestimulus attention blocks and 9 fixation blocks. To increase design efficiency, auditory and visual spatial locations and response modality were pseudo-randomised across trials. Throughout the task, participants fixated a cross (1° diameter) in the centre of the screen. On each keypad, a specific key corresponded to one of the three signal locations along the azimuth. Participants reported their perceived signal location in the sensory modality indicated by the postcue as accurately as possible using the corresponding keypad. The mapping of hands (left/right), report modalities (auditory/visual), and colours of the letter and fixation crosses (blue/yellow) was counterbalanced within participants across days. At the beginning of each day (both for psychophysics and fMRI), participants were familiarised with the stimuli and procedure via one preliminary practice run. For the psychophysics experiment outside the scanner, every participant completed 3 runs in 1 day (6 trials / condition / run × 36 conditions × 3 runs = 648 trails in total). For the fMRI experiment, every participant completed 14 scanning runs over the course of 4 days (6 trials / condition / run × 36 conditions × 14 runs = 3,024 trails in total).

### Experimental setup—Psychophysics experiment

The experiment was presented via Psychtoolbox version 3.0.11 [75] running under MATLAB R2014a (MathWorks, USA) on a Windows machine (Microsoft 7 2009, USA). Auditory stimuli were presented with headphones (HD 280 PRO, Sennheiser, Germany). Visual stimuli were presented on a Gamma-calibrated LCD monitor (30” Dell UltraSharp U3014, USA; 2,560 × 1,600 pixels resolution; 60-Hz frame rate). We adjusted audiovisual latencies in the presentation software and confirmed their synchrony by recording and measuring their relative latencies using a microphone and a photodiode. To mimic the sensory environment in the MRI experiment, scanner noise was played at 80dB SPL through external loudspeakers positioned at each side of the monitor. Participants sat in a dimly lit cubicle in front of the computer monitor at a viewing distance of 50 cm with their head positioned on a chin rest. They gave responses via two keypads (Targus, USA), one per hand, and report modality. Gaze position was monitored via Tobii EyeX eyetracking system (Tobii, Sweden).

### Experimental setup—fMRI experiment

The experiment was presented via Psychtoolbox version 3.0.11 [75] running under MATLAB R2011b (MathWorks) on a MacBook Pro (Mac OSX 10.6.8). Auditory stimuli were played using MR-compatible headphones (MR Confon HP-VS03). Visual stimuli were back-projected onto a Plexiglas screen using a Barco Present-C F-Series projector (F35 WUXGA, 1,280 × 1,024 pixels resolution; 60-Hz frame rate), and they were visible to the participants via a mirror mounted on the MR head coil (horizontal visual field of approximately 40° visual angle at a viewing distance of approximately 68 cm). Participants gave responses via two MR-compatible keypads (NATA LXPAD 1×5-10M, NATAtech.com), one per hand, and report modality.

### MRI data acquisition

A 3T Philips Achieva MR scanner was used to acquire both a T1-weighted anatomical image (TR = 8.4 ms, TE = 3.8 ms, flip angle = 8°, FOV = 288 mm × 232 mm, 175 sagittal slices acquired in sequential ascending direction, voxel size = 1 × 1 × 1 mm^3^) and T2*-weighted axial echoplanar images (EPI) with blood oxygen level–dependent contrast (gradient echo, SENSE factor of 2, TR = 2800 ms, TE = 40 ms, flip angle = 90°, FOV = 192 × 192 × 114 mm^2^, 38 axial slices acquired in sequential ascending direction, voxel size = 2.5 × 2.5 × 2.5 mm^3^ + 0.5 mm interslice gap). For each participant, a total of 276 volumes × 14 runs were acquired, after discarding the first 4 volumes of each run to allow for T1 equilibration effects. Data acquisition was performed over the course of 4 days, and the anatomical image was acquired at the end of the last day.

### Behavioural data analysis—Psychophysics and fMRI experiments

For both the psychophysics experiment outside the scanner and the fMRI experiment, we limited the analyses of behavioural responses to trials without missed responses (i.e., no answer within 2-second response time window) or premature responses (i.e., response times <100 ms). Further, we did not include any trials where observers used the wrong keypad indicating that they were confused about the sensory modality of the signal location they had to report. Only few trials were discarded for the psychophysics experiment (3.4% ± 0.7% [across participants mean ± SEM]) and the fMRI experiment (3.0% ± 1.0% [across participants mean ± SEM]). For psychophysics outside the scanner, we also excluded trials without central fixation during stimuli presentation. Saccades were defined as eye movements outside a 1.3° circular area centred on participant’s median of fixation determined in calibration trials [69]. Participants successfully maintained fixation with only a small number of rejected trials (0.4% ± 0.1% [across participants mean ± SEM]).

In the following, we describe the analysis of (i) behavioural response times; (ii) the audiovisual weight index; and (iii) the Bayesian modelling analysis that were applied to both the psychophysics and the fMRI experiments.

### General linear model–based analysis of response times

For each experimental trial, we computed response times from the onset of the report cue (Fig 2B). For each participant, median response times of each experimental condition were averaged across all combinations of AV locations at a particular level of absolute AV spatial disparity and entered into a 2 (prestimulus attention: auditory/visual) × 2 (poststimulus report: auditory/visual) × 3 (AV spatial disparity: 0°, 9°, or 18° visual angle, i.e., none, low, or high disparity) repeated measures ANOVA. We present two-tailed *p*-values for the statistical comparisons of interest in the main text (i.e., cue validity effect, spatial congruency effect, interaction between spatial congruency, and poststimulus report). All statistical results are comprehensively summarised in S2 Table.

### Analysis of audiovisual weight index w_AV_

To assess the influence of the auditory and visual signal locations on observers’ reported auditory (or visual) locations, we computed an audiovisual weight index *w*_*AV*_ for the spatially incongruent trials (i.e., AV spatial disparity greater than 0). The *w*_*AV*_ index is defined as the difference between the reported location and the auditory location, scaled by the difference between the reported visual and auditory locations on audiovisual congruent trials. The denominator acts as a scaling operator for all conditions at a particular level of spatial disparity; to increase its estimation precision, we computed the denominator pooled over all prestimulus attention and poststimulus report conditions across all participants. Hence, we obtained 3 denominators (8.40 for −9° versus 0°; 8.65 for 0 versus 9°; and 17.05 for −9° versus 9° visual angle) using the across participants’ and across (prestimulus × poststimulus attention) conditions’ mean reported locations of the congruent conditions.

$$w_{AV,XY}=\frac{Reported\;location_{Incongruent,\;A=X,\;V=Y}-Reported\;location_{Congruent,\;AV=X}}{Reported\;location_{Congruent,\;AV=Y}-Reported\;location_{Congruent,\;AV=X}}$$ (1)

Under the assumption of comparable central biases across all conditions, an audiovisual weight index *w*_*AV*_ of one indicates that observer’s spatial report relies completely on the visual signal location. An audiovisual weight index *w*_*AV*_ of 0 indicates that observer’s spatial report relies completely on the auditory signal location. We averaged the *w*_*AV*_ index across all combinations of AV locations at a particular level of absolute AV spatial disparity. We thus analysed the audiovisual weight index in a 2 (prestimulus attention: auditory versus visual) × 2 (poststimulus report: auditory versus visual) × 2 (AV spatial disparity: 9° or 18° visual angle, i.e., low or high disparity) factorial design. Please note that Bayesian models were fitted directly to observers’ responses across the 36 conditions in our original 2 (prestimulus attention: auditory versus visual) × 2 (poststimulus report: auditory versus visual) × 3 (A location: −9°, 0°, and 9° visual angle) × 3 (V location: −9°, 0°, and 9° visual angle) design.

To refrain from making any parametric assumptions, we evaluated the main effects of prestimulus attention, poststimulus report, AV spatial disparity, and their interactions in this factorial design using two-tailed (unless otherwise stated) permutation testing at the between-subject level (4,096 cases with *n* = 12). For effect sizes, we report the difference of the across participants’ mean empirical effect and the mean of the nonparametric null distribution and 95% CIs.

### Bayesian modelling

Combining Bayesian modelling and psychophysics, we assessed whether prestimulus attention and poststimulus report influence multisensory estimates via different computational mechanisms. In the following, we will first describe the Bayesian causal inference model from which we will then derive the forced fusion model as a special case (details can be found in [20,27]). In a second step, we will then describe how prestimulus attention and poststimulus report may affect multisensory processing in these models.

The Bayesian causal inference model (Fig 1A and 1B) describes how observers should combine information from different senses when there is uncertainty about whether signals are caused by common or separate sources. Briefly, the generative model of Bayesian causal inference explicitly models the two potential causal structures, i.e., common source or separate sources, which could have generated the sensory signals. It assumes that common (*C* = 1) or independent (*C* = 2) causes are sampled from a binomial distribution defined by the common cause prior *P*_*common*_. For a common source, the “true” location *S*_*AV*_ is drawn from the spatial prior distribution *N*(*μ*_*AV*_,*σ*_*P*_). For two independent causes, the “true” auditory (*S*_*A*_) and visual (*S*_*V*_) locations are drawn independently from this spatial prior distribution. For the spatial prior distribution, we assume a central bias (i.e., *μ* = 0). We introduce sensory noise by drawing *x*_*A*_ and *x*_*V*_ independently from normal distributions centred on the true auditory (resp. visual) locations with parameters $\sigma_{A}^{2}$ (resp. $\sigma_{V}^{2}$). Thus, the generative model (i.e., without attentional effects) includes the following four free parameters: the causal prior *P*_*common*_, the spatial prior variance $\sigma_{P}^{2}$, the auditory variance $\sigma_{A}^{2}$, and the visual variance $\sigma_{V}^{2}$.

The posterior probability of the underlying causal structure is inferred by combining the common source prior with the sensory evidence according to Bayes’ rule:

$$p\left(C=1|x_{A},x_{V}\right)=\frac{p(x_{A},x_{V}|C=1)P_{common}}{p\left(x_{A},x_{V}\right)}$$ (2)

In the case of a common source (*C* = 1), the estimate of the audiovisual location is obtained by averaging the auditory and visual estimates along with the spatial prior weighted by their relative reliabilities (i.e., “forced fusion estimate”):

$${\hat{S}}_{AV,C=1}=\frac{x_{A}/\sigma_{A}^{2}+x_{V}/\sigma_{V}^{2}+\mu_{P}/\sigma_{P}^{2}}{1/\sigma_{A}^{2}+1/\sigma_{V}^{2}+1/\sigma_{P}^{2}}$$ (3)

In the case of independent sources (*C* = 2), the auditory and visual stimulus locations are estimated independently (i.e., “unisensory auditory or visual full segregation estimates”):

$${\hat{S}}_{A,C=2}=\frac{x_{A}/\sigma_{A}^{2}+\mu_{P}/\sigma_{P}^{2}}{1/\sigma_{A}^{2}+1/\sigma_{P}^{2}},\;{\hat{S}}_{V,C=2}=\frac{x_{V}/\sigma_{V}^{2}+\mu_{P}/\sigma_{P}^{2}}{1/\sigma_{V}^{2}+1/\sigma_{P}^{2}}$$ (4)

To provide a final estimate of the auditory and visual locations, the brain can combine the estimates from the two causal structures using various decision functions [27]. According to the “model averaging” strategy, a final “Bayesian causal inference estimate” is obtained by combining the integrated forced fusion estimate with the task-relevant unisensory (i.e., either auditory or visual) full segregation estimate weighted in proportion to the posterior probability of the respective causal structures:

$${\hat{S}}_{A}=p\left(C=1|x_{A},x_{V}\right){\hat{S}}_{AV,C=1}+(1-p\left(C=1|x_{A},x_{V})\right){\hat{S}}_{A,C=2}$$ (5)

$${\hat{S}}_{V}=p\left(C=1|x_{A},x_{V}\right){\hat{S}}_{AV,C=1}+(1-p\left(C=1|x_{A},x_{V})\right){\hat{S}}_{V,C=2}$$ (6)

The forced fusion model can be considered a special case of Bayesian causal inference, in which it is known without uncertainty that the two signals come from one common source (i.e., causal prior *P*_*common*_ = 1). In this “forced fusion scenario”, auditory and visual signals are integrated weighted by their relative reliabilities as described by Eq (3). The forced fusion model includes only three free parameters: the variances of the auditory signal, the visual signal, and the spatial prior.

How can prestimulus attention and poststimulus report affect multisensory inference in the forced fusion and Bayesian causal inference model (Fig 1C)? Prestimulus attention may affect the reliabilities of the sensory signals prior to integration both in the forced fusion and the Bayesian causal inference model. For example, we would expect prestimulus attention to vision to increase the reliability (i.e., decrease the variance) of the visual information and thereby increase its weight in the fusion process (and vice versa for prestimulus attention to audition). Likewise, poststimulus report may affect the reliabilities of the sensory signals prior to integration in the forced fusion model (or the fusion component in the causal inference model). In this case, prestimulus attention and poststimulus report would alter audiovisual integration via the same computational mechanisms. While the effect of prestimulus attention on sensory variance seems intuitive, a corresponding effect of poststimulus report may be less plausible. For poststimulus report to affect sensory variances, observers would need to keep unisensory representations separate until the presentation of the poststimulus cue. Further, one would need to assume that poststimulus report can modify the reliability of sensory representations even after stimulus offset. For instance, poststimulus report may influence how higher-order areas internally sample or accumulate sensory information prior to integration. Nevertheless, it seems less likely that poststimulus report alters the reliability of sensory representations per se.

Alternatively, the effect of poststimulus report may arise naturally from Bayesian causal inference. Here, the brain reads out a final estimate that flexibly combines the forced fusion estimate with the auditory or visual segregation estimates weighted by the posterior probability of the respective causal structures. In this latter case, poststimulus report would not influence the sensory reliabilities prior to fusion, but only the flexible readout of the final perceptual estimate according to current task demands.

Please note that both mechanisms would induce a difference in the audiovisual weight index *w*_*AV*_ for auditory and visual poststimulus report that is directly computed from observers’ localisation responses. However, only for the latter mechanism, i.e., the flexible readout of the task-relevant estimate, would this difference between auditory and visual poststimulus report increase with audiovisual spatial disparity [11,12,47]. This is because greater audiovisual disparity increases the posterior probability of separate causes, which, in turn, increases the weight for the segregated estimates that are selectively read out.

To arbitrate between these different hypotheses, we used Bayesian model comparison in a 2 × 3 factorial model space. Along factor 1, we assessed the influence of pre-stimulus attention comparing models in which the auditory and visual variances were i. fixed or ii. allowed to differ between auditory and visual attention (i.e. auditory variance: $\sigma_{A\;attA}^{2}$, $\sigma_{A\;attV}^{2}$; visual variance: $\sigma_{V\;attA}^{2}$, $\sigma_{V\;attV}^{2}$). Along factor 2, we assessed the impact of post-stimulus report by comparing i. a forced fusion model in which the sensory variances were fixed, ii. a forced fusion model in which the sensory variances were allowed to differ between auditory and visual post-stimulus report (i.e. auditory variance: $\sigma_{A\;repA}^{2}$, $\sigma_{A\;repV}^{2}$; visual variance: $\sigma_{V\;repA}^{2}$, $\sigma_{V\;repV}^{2}$) and iii. a Bayesian causal inference model in which the influence of post-stimulus report arises via a late flexible read-out.

In total, we thus compared 6 models: (i) Forced Fusion, Attention + Report fixed, with 3 parameters (A and V variances, spatial prior); (ii) Forced Fusion, Attention flexible + Report fixed, with 5 parameters (2 A and 2 V variances, spatial prior); (iii) Forced Fusion, Attention fixed + Report flexible, with 5 parameters (2 A and 2 V variances, spatial prior); (iv) Forced Fusion, Attention flexible + Report flexible, with 9 parameters (4 A and 4 V variances, spatial prior); (v) Bayesian causal inference with 4 parameters (A and V variances, spatial prior, causal prior); and (vi) Bayesian causal inference, Attention flexible, with 6 parameters (2 A and 2 V variances, spatial prior, causal prior).

We fitted each model individually (i.e., separately for each participant) to participants’ behavioural localisation responses based on the predicted distributions of the spatial estimates (i.e., $p(\hat{S}|S_{A},S_{V})$; we use $\hat{S}$ as a variable to refer generically to any (i.e., auditory or visual or audiovisual) spatial estimate for each combination of auditory (*S*_*A*_) and visual (*S*_*A*_) source locations. To marginalise over the internal variables *x*_*A*_ and *x*_*V*_ that are not accessible to the experimenter, the predicted distributions were generated by simulating *x*_*A*_ and *x*_*V*_ 10,000 times for each of the 3 (auditory location) × 3 (visual location) × 2 (prestimulus attention to vision versus audition) × 2 (poststimulus report of vision versus audition) conditions and inferring a spatial estimate $\hat{S}$ from Eqs (2–6) [20] for each simulated *x*_*A*_ and *x*_*V*_. To link any of those $p(\hat{S}|S_{A},S_{V})$ to participants’ auditory and visual discrete localisation responses at the behavioural level, we assumed that participants selected the button that is closest to $\hat{S}$ and binned the $\hat{S}$ accordingly into histograms for each condition (with three bins corresponding to the three buttons). Thus, we obtained a histogram of predicted localisation responses for each model separately for each condition and individually for each participant. Based on these histograms, we computed the probability of a participant’s counts of localisation responses using the multinomial distribution [20]. This provides the likelihood of the model given participants’ response data. Assuming independence of conditions, we summed the log likelihoods across conditions. To obtain maximum likelihood estimates for the parameters of the models, we used a nonlinear simplex optimisation algorithm as implemented in MATLAB’s *fminsearch* function (MATLAB R2016a). This optimisation algorithm was initialised with a parameter setting that obtained the highest log likelihood in a prior grid search. The model fit for behavioural data was assessed by the coefficient of determination *R*^2^ [76] defined as

$$R^{2}=1-\exp-\frac{2}{n}\left(\text{l}(\hat{ß})-\text{l}\left(0\right)\right)$$ (7)

where l($\hat{ß}$) and l(0) denote respectively the log likelihoods of the fitted and the null model, and *n* is the number of data points. For the null model, we assumed that an observer randomly chooses one of the three response options, i.e. we assumed a discrete uniform distribution with a probability of 0.33. As in our case the Bayesian causal inference model’s responses were discretized to relate them to the three discrete response options, the coefficient of determination was scaled (i.e., divided) by the maximum coefficient (cf.[76]) defined as:

$$\max\left({\text{R}}^{2}\right)=1-\text{exp}\left(\frac{2}{\text{n}}\text{l}\left(0\right)\right)$$ (8)

To identify the optimal model for explaining participants’ data (i.e., localisation responses at the behavioural level), we compared the candidate models using the Bayesian information criterion (BIC) as an approximation to the log model evidence[77]:

$$BIC=\;-\text{ln}\;\hat{L}+k*\text{ln}\;n,$$ (9)

where $\hat{L}$ denotes the likelihood, *n* the number of data points and *k* the number of parameters. Importantly, the BIC depends on both model complexity and model fit. We performed Bayesian model selection[78] at the group (i.e. random effects) level as implemented in SPM12[79] to obtain the protected exceedance probability that one model is better than any of the other candidate models above and beyond chance. Further, for the winning model (i.e. the Bayesian causal inference model with $\sigma_{A}^{2}$ and $\sigma_{V}^{2}$ differing between auditory and visual pre-stimulus attention) we evaluated pair-wise changes in variance as a function of pre-stimulus attention via one-tailed permutation testing, based on our a-priori hypotheses (i.e. auditory variance $\sigma_{A}^{2}$ decreases for auditory relative to visual pre-stimulus attention; vice versa, visual variance $\sigma_{V}^{2}$ decreases for visual relative to auditory pre-stimulus attention). For effect sizes, we report the difference of the across-participants mean empirical effect and the mean of the non-parametric null-distribution, and 95% confidence intervals.

### Neuroimaging data analysis

#### Univariate analysis

The functional MRI data were analysed with statistical parametric mapping (SPM12; Wellcome Department of Imaging Neuroscience, London; www.fil.ion.ucl.ac.uk/spm [79]). Scans from each participant were realigned using the first as a reference, unwarped, slice-time corrected, and spatially normalised into MNI standard space using parameters from segmentation of the T1 structural image, resampled to a spatial resolution of 2 × 2 × 2 mm^3^ and spatially smoothed with a Gaussian kernel of 8-mm full width at half maximum. The time series of all voxels were high-pass filtered to 1/128 Hz. The fMRI experiment was modelled in an event-related fashion with regressors entered into the design matrix after convolving each event-related unit impulse with a canonical hemodynamic response function and its first temporal derivative. In addition to modelling the 36 experimental conditions in our 2 (prestimulus attention: auditory versus visual) × 2 (poststimulus report: auditory versus visual) × 3 (visual location) × 3 (auditory location) factorial design, the statistical model included the onsets of the “attention” precue (i.e., at the beginning of each block) as a separate regressor. Nuisance covariates included the realignment parameters to account for residual motion artefacts. In a control analysis, we replicated the current findings by also modelling as one single extra regressor all missed or premature responses or responses with the wrong keypad (hence, we do not report this in details). Condition-specific effects (i.e., parameter estimates for the canonical hemodynamic response function regressors) for each participant were estimated according to the general linear model and passed to a second-level analysis as contrasts. This involved creating 36 contrast images (i.e., each of the 36 conditions relative to fixation, summed over the 14 runs) for each participant and entering them into a second-level ANOVA. Inferences were made at the second level to allow a random-effects analysis and inferences at the population level [79].

In the main manuscript, we present the cue invalidity effects (i.e., prestimulus attention × poststimulus report interaction: [attVrepA & attArepV] > [attArepA & attVrepV]), identifying stronger activations for trials with invalid than valid precues. Further, we tested for audiovisual spatial incongruency effects (i.e., [AVincongruent (AV disparity ≠ 0°) > AVcongruent (AV disparity = 0°)]), i.e., identifying stronger activations for AV incongruent than congruent stimuli. Together, these contrasts allowed us to test the hypothesis that the same widespread frontoparietal insular system (previously implicated in cognitive control and selective attention [46]) is activated when the brain detects spatial conflicts between audiovisual signals and conflicts between pre- and postcues. For comprehensive characterisation of the data, S3 and S4 Tables also report the opposite contrasts, i.e., the cue validity effect (i.e., [attVrepA & attArepV] < [attArepA & attVrepV]) and the audiovisual congruency effect (i.e., [AVincongruent (AV disparity ≠ 0°) < AVcongruent (AV disparity = 0°)]). Finally, we report the cue invalidity effects separately for auditory (i.e., attVrepA > attArepA) and visual (i.e., attArepV > attVrepV) reports in S5 Table, the main effect of prestimulus attention (i.e., [attA > attV] and vice versa), and the main effect of poststimulus report (i.e., [repA > repV] and vice versa) in S6 Table. Unless otherwise stated, we report activations at *p* < 0.05 at the peak level family-wise error (FWE)-corrected for multiple comparisons within the entire brain.

#### Multivariate decoding and neural audiovisual weight index nw_AV_

For the multivariate pattern analysis, scans from each participant were realigned using the first as a reference, unwarped, and slice-time corrected. The time series of all voxels were high-pass filtered to 1/128 Hz. All data were analysed in native participant space and without any smoothing using the same design matrix as in the univariate analysis, except that we concatenated pairs of successive runs to double the number of trials for each condition and hence increase the signal-to-noise ratio. The voxel-wise magnitude of the BOLD signal in response to the audiovisual onsets was defined by the parameter estimates pertaining to the canonical hemodynamic response function. Each parameter estimate image was based on 12 trials, and there were 7 parameter estimate images per condition (i.e., 1 parameter estimate per condition and run, 7 runs). BOLD response patterns were extracted from five a priori ROIs along the visual and auditory dorsal hierarchies (Fig 4A; see section “Regions of interest definition”). The resulting voxel response patterns were scaled to the range 0 to 1 for each ROI (i.e., “image scaling”). Multivariate decoding was performed using The Decoding Toolbox 3.96 (TDT) [80]. For each participant and ROI, we employed a linear SVR model as implemented in LIBSVM 3.17[81] (C = 1 and nu = 0.5). The SVR model was trained to learn the mapping from the fMRI activation vectors to the external spatial locations based on the audiovisually congruent conditions (including conditions of auditory and visual prestimulus attention and poststimulus report) from all but one run (7 runs in total). This learnt mapping was then used to decode the spatial location from the fMRI activation vectors of the spatially congruent and incongruent audiovisual conditions of the remaining run (Fig 4B). In a leave-one-run-out cross-validation scheme, the training test procedure was repeated for all 7 runs.

We then asked two questions. First, we investigated whether the true signal location of the audiovisual congruent signals can be decoded successfully from the fMRI activation patterns in our ROIs. We evaluated decoding accuracy in terms of the Pearson correlation between the true and the decoded spatial locations in audiovisual congruent conditions alone (n.b. we can compute audiovisual accuracy in a meaningful way only for the congruent audiovisual trials). Individual correlation coefficients were Fisher-z transformed and then tested against 0 (i.e., r > 0) via 1-tailed permutation testing at the between-subject level. Alongside significance results, we report the inverse-transformed across participants’ mean and 95% CIs. For effect sizes, we report the difference of the across participants’ mean empirical effect and the mean of the nonparametric null distribution, and 95% CIs. It is important to emphasise that comparison of decoding accuracy across regions should be made with great caution. This is because the ability to decode information from fMRI responses depends on several factors including whether information is neural encoding, its representational format, and a region’s vascular organisation. Most notably, spatial information is represented topographically in visual cortices [82], but via rate-based code in auditory cortices [59,60].

Second, we investigated how the five ROIs in the visual and auditory processing hierarchies integrate auditory and visual signals into spatial representations. For this, we focused on the decoded spatial locations for the spatially incongruent conditions, which provide information about how a brain region combines visual and auditory spatial signals into spatial representations. We quantified the influence of the auditory and visual signal location on the decoded spatial estimates for each ROI using the neural audiovisual weight index *nw*_*AV*_, which is defined similarly as in the behavioural analysis:

$$nw_{AV,XY}=\frac{Decoded\;location_{Incongruent,\;A=X,\;V=Y}-Decoded\;location_{Congruent,\;AV=X}}{Decoded\;location_{Congruent,\;AV=Y}-Decoded\;location_{Congruent,\;AV=X}}$$ (10)

Please note that the denominator controls at least partly for differences in decodability between areas. Similar to our behavioural analysis, we increased the estimation precision of the denominator by pooling over all prestimulus attention × poststimulus report conditions × runs across all participants. First, we averaged the locations decoded from the 28 parameter estimates (i.e., 2 prestimulus attention × 2 poststimulus report × 7 runs) for each congruent location within each participant and then formed the across participants’ mean. Second, we formed three across participants’ mean denominators by forming the appropriate differences in locations decoded from the congruent conditions for the three levels of spatial disparity (i.e., for −9° versus 0°; for 0 versus 9°; and for −9° versus 9° visual angle). Under the assumption of comparable central biases across all conditions, the *nw*_*AV*_ should be equal to 1 in regions that encode the location of the visual signal irrespective of the auditory signal, i.e., formally,

$$Decoded\;location_{Incongruent,\;A=X,\;V=Y}=\;Decoded\;location_{Congruent,\;AV=Y}$$ (11)

Conversely, the *nw*_*AV*_ should be equal to 0 in regions that encode the location of the auditory signal irrespective of the visual signal, i.e., formally,

$$Decoded\;location_{Incongruent,\;A=X,\;V=Y}=\;Decoded\;location_{Congruent,\;AV=X}$$ (12)

We divided the decoded biases (Eqs 11 and 12) by the corresponding spatial disparities to obtain *nw*_*AV*_ index. We averaged the *nw*_*AV*_ indices across all combinations of AV locations at a particular level of absolute AV spatial disparity.

First, we assessed in low-level visual (resp. auditory) cortices whether the audiovisual weight index is significantly different from 1 (resp. 0) indicating significant crossmodal influences at the early cortical level. Second, we evaluated the main effects of prestimulus attention, poststimulus report, AV spatial disparity, and their interactions in the factorial design. To refrain from making any parametric assumptions, we assessed all these effects using permutation testing as described under the behavioural analysis.

### Regions of interest definition

Our ROI analysis was performed in accordance with our previous publications [7,11,12]. All ROIs were defined bilaterally, i.e., via combination of corresponding areas from left and right hemispheres. In line with these previous studies [7,11,12], we focused our analysis on a specific set of visual and auditory ROIs along the auditory and visual spatial processing hierarchies [83]: V1-3 (2385 voxels), pIPS (1012 voxels), aIPS (580 voxels), low-level auditory cortices (A; 226 voxels), and hA (359 voxels). Visual ROIs were defined using volume-based probability maps from a probabilistic atlas for visual topography [82]. V1-3 comprised ventral and dorsal areas V1-3; pIPS comprised areas IPS0, IPS1, and IPS2; and aIPS comprised areas IPS3, IPS4, and SPL1 [82]. Low-level auditory cortex (A) comprised areas TE1.0 and 1.1 from the Anatomy Toolbox [84]. hA comprised planum temporale and transverse temporal sulcus from the Destrieux atlas (2009) of Freesurfer 5.3.0 [85].

## Supporting information

S1 Data ZIP file containing dataset underlying Fig 2C and 2D and S3–S6 Tables. The data are stored in MATLAB structures.
(ZIP)

S2 Data ZIP file containing datasets underlying Figs 3 and 4 and S2 and S1, S7, S9 and S10 Tables. The data are stored in MATLAB structures.
(ZIP)

S3 Data ZIP file containing dataset underlying S1B Fig and S11 Table. The data are stored in MATLAB structures.
(ZIP)

S1 Text Unisensory auditory localisation inside the scanner.
(DOCX)

S1 Table Response times in the psychophysics and fMRI experiments. Across participants’ mean (±SEM) as a function of prestimulus attention (attA, auditory; attV, visual), poststimulus report (repA: auditory; repV: visual), and audiovisual spatial disparity (dispN: no disparity; dispL: low; dispH: high).
(DOCX)

S2 Table Statistical results of response times in the psychophysics and fMRI experiments. Main effects and interactions of the 2 (prestimulus attention, Att: attA, attV) × 2 (poststimulus report, Rep: repA, repV) × 3 (audiovisual spatial disparity, Disp: low, high) repeated measures ANOVA. Greenhouse–Geisser correction is applied to degrees of freedom (df1 and df2) in case of violation of sphericity (Mauchly test).
(DOCX)

S3 Table fMRI univariate results: Audiovisual spatial (in)congruency. Effect of audiovisual spatial incongruency [AVincongruent (AV disparity ≠ 0°) > AVcongruent (AV disparity = 0°)] and congruency [AVcongruent (AV disparity = 0°) > AVincongruent (AV disparity ≠ 0°)]. *p*-Values are FWE corrected at the peak level for multiple comparisons within the entire brain. FWE, family-wise error; L, left; R, right.
(DOCX)

S4 Table fMRI univariate results: Cue (in)validity. Effect of cue invalidity [Invalid (attVrepA & attArepV) > Valid (attArepA & attVrepV)] and validity [Valid (attArepA & attVrepV) > Invalid (attVrepA & attArepV)], where attA: auditory prestimulus attention; attV: visual prestimulus attention; repA: auditory poststimulus report; repV: visual poststimulus report. *p*-Values are FWE corrected at the peak level for multiple comparisons within the entire brain. FWE, family-wise error; L, left; R, right.
(DOCX)

S5 Table fMRI univariate results: Cue invalidity separately for auditory and visual report. Effect of cue invalidity separately for auditory (attVrepA > attArepA) and visual report (attArepV > attVrepV), where attA: auditory attention; attV: visual attention; repA: auditory report; repV: visual report. *p*-Values are FWE corrected at the peak level for multiple comparisons within the entire brain. FWE, family-wise error; L, left; R, right.
(DOCX)

S6 Table fMRI univariate results: Poststimulus report and prestimulus attention. Effect of auditory relative to visual report (repA > repV) and vice versa (repV > repA); effect of auditory relative to visual attention (attA > attV) and vice versa (attV > attA). *p*-Values are FWE corrected at the peak level for multiple comparisons within the entire brain. FWE, family-wise error; L, left; R, right.
(DOCX)

S7 Table Audiovisual weight index (*w*_*AV*_) in the psychophysics and fMRI experiments and neural audiovisual weight index (*nw*_*AV*_) for each ROI. **(a)** Across participants’ mean (±SEM) *w*_*AV*_ as a function of prestimulus attention (attA, auditory; attV, visual), poststimulus report (repA: auditory; repV: visual) and audiovisual spatial disparity (dispL: low; dispH: high). **(b)** Across participants’ mean (±SEM) *nw*_*AV*_ as a function of prestimulus attention (attA, auditory; attV, visual) and poststimulus report (repA: auditory; repV: visual) for each ROI. A, low-level auditory cortex; aIPS, anterior intraparietal sulcus; hA, higher-order auditory cortex; pIPS, posterior intraparietal sulcus; ROI, region of interest; V1-3, low-level visual cortex.
(DOCX)

S8 Table Statistical significance (*p*-value and effect size with 95% CI) of behavioural audiovisual weight index (*w*_*AV*_) and of neural audiovisual weight index (*nw*_*AV*_) for each ROI. Main effects and interactions for **(a)** behavioural audiovisual weight index (*w*_*AV*_) in the psychophysics and fMRI experiments and **(b)** neural audiovisual weight index (*nw*_*AV*_) in the 2 (prestimulus attention, Att: attA, attV) × 2 (poststimulus report, Rep: repA, repV) × 2 (audiovisual spatial disparity, Disp: low, high) factorial design. *p*-Values are based on two-tailed permutation tests apart from those for main effect of prestimulus attention (Att: attV > attA) and poststimulus report (Rep: repV > repA), which are one-tailed because of a priori hypotheses. Effect sizes [95% CI] correspond to the difference of the across participants’ mean empirical effect and the mean of the nonparametric null distribution. A, low-level auditory cortex; aIPS, anterior intraparietal sulcus; hA, higher-order auditory cortex; pIPS, posterior intraparietal sulcus; ROI, region of interest; V1-3, low-level visual cortex.
(DOCX)

S9 Table Proportion of correct responses in the psychophysics and fMRI experiments. Across participants’ mean (±SEM) as a function of prestimulus attention (attA, auditory; attV, visual), poststimulus report (repA: auditory; repV: visual) and audiovisual spatial disparity (dispN: no disparity; dispL: low; dispH: high).
(DOCX)

S10 TableBayesian modelling results in the psychophysics and fMRI experiments.Using Bayesian model comparison, we assessed the influence of prestimulus attention and poststimulus report in a 2 × 3 factorial model space. Along the first factor, we assessed the influence of prestimulus attention comparing models in which the auditory and visual variances were (i) constant (Att fixed: $\sigma_{A\;attA}^{2}$ = $\sigma_{A\;attV}^{2}$, $\sigma_{V\;attA}^{2}$ = $\sigma_{V\;attV}^{2}$); or (ii) different (Att free: $\sigma_{A\;attA}^{2}$, $\sigma_{A\;attV}^{2}$, $\sigma_{V\;attA}^{2}$, $\sigma_{V\;attV}^{2}$) across prestimulus attention. Along the second factor, we assessed the influence of poststimulus report by comparing (i) an FF model in which the sensory variances were fixed (Rep fixed: $\sigma_{A\;repA}^{2}$ = $\sigma_{A\;repV}^{2}$, $\sigma_{V\;repA}^{2}$ = $\sigma_{V\;repV}^{2}$); (ii) an FF model in which the sensory variances were allowed to differ between auditory and visual report (Rep free: $\sigma_{A\;repA}^{2}$, $\sigma_{A\;repV}^{2}$, $\sigma_{V\;repA}^{2}$, $\sigma_{V\;repV}^{2}$); and (iii) a BCI model in which the influence of poststimulus report arises via a late flexible readout. We report across participants’ mean (±SEM) of the models parameters: *P*_*common*_, prior common source probability; *σ*_*P*_, spatial prior standard deviation (° visual angle); *σ*_*A*_, auditory likelihood standard deviation (° visual angle); *σ*_*V*_, visual likelihood standard deviation (° visual angle). In addition, *R*^2^, coefficient of determination; *relBIC*, BIC of a model summed over participants (BIC = LL − 0.5 × P × ln(N), LL = log-likelihood, P = number of parameters, *N* = number of data points) relative to the “BCI Att free” model (a model with smaller relBIC provides better data explanation); pEP, protected exceedance probability (probability that a model is more likely than the other models, beyond differences due to chance). BCI, Bayesian causal inference; FF, forced fusion.(DOCX)

S11 Table fMRI results of unisensory auditory localisation inside the scanner. Effect of auditory localisation collapsing across spatial locations (Task > Baseline) and separately for left versus right lateralised sounds (SoundL > SoundR; SoundR > SoundL). *p*-Values are FWE corrected at the peak level for multiple comparisons within the entire brain. FWE, family-wise error; L, left; R, right.
(DOCX)

S1 Fig fMRI results of unisensory auditory localisation inside the scanner. **(a)** Experimental procedure: after each sound presentation, participants reported their perceived auditory location via button press with the correspondent key. **(b)** Increases of BOLD response for lateralised right versus left sounds (blue) and vice versa (orange) are rendered on an inflated canonical brain (*p* < 0.001 uncorrected at peak level for visualisation purposes, extent threshold k > 0 voxels). Bar plots represent across participants’ mean (±SEM) parameter estimates in nondimensional units (corresponding to percentage whole-brain mean) from left (x = −50, y = −32, z = 8) and right (x = 52, y = −22, z = 4) plana temporalia. The data used to make this figure are available in S3 Data. C, centre; L, left; R, right.
(TIF)

S2 Fig Distributions of spatial estimates in the fMRI experiment. The distribution of spatial estimates (across participants’ mean) given by observers’ behavioural localisation responses (solid lines) or predicted by the BCI model with attentional effects (i.e., “BCI model, Att free”) fitted to observers’ behavioural responses (dashed lines) are shown across all conditions in our a 3 (auditory location) × 3 (visual location) × 2 (prestimulus attention: auditory, visual) × 2 (poststimulus report: auditory, visual) factorial design. The data used to make this figure are available in S2 Data. BCI, Bayesian causal inference.
(TIF)

## Abbreviations

aIPS: anterior intraparietal sulcus
BIC: Bayesian information criterion
BOLD: blood-oxygen-level dependent
EEG: electroencephalography
fMRI: functional magnetic resonance imaging
FWE: family-wise error
hA: higher-order auditory cortex
HRTF: head-related transfer function
pIPS: posterior intraparietal sulcus
RMSE: root mean square error
ROI: region of interest
SVR: support vector regression
V1-3: low-level visual cortex

## References

[1] Alais D, Burr D. The ventriloquist effect results from near-optimal bimodal integration. Curr Biol. 2004;14(3):257–62. doi: 10.1016/j.cub.2004.01.029 14761661

[2] Ernst MO, Banks MS. Humans integrate visual and haptic information in a statistically optimal fashion. Nature. 2002;415(6870):429–33. doi: 10.1038/415429a 11807554

[3] Ernst MO, Bülthoff HH. Merging the senses into a robust percept. Trends Cogn Sci. 2004;8(4):162–9. doi: 10.1016/j.tics.2004.02.002 15050512

[4] Fetsch CR, Pouget A, DeAngelis GC, Angelaki DE. Neural correlates of reliability-based cue weighting during multisensory integration. Nat Neurosci. 2012;15(1):146–54. 10.1038/nn.2983 PMC3398428 22101645

[5] Fetsch CR, DeAngelis GC, Angelaki DE. Bridging the gap between theories of sensory cue integration and the physiology of multisensory neurons. Nat Rev Neurosci. 2013;14(6):429–42. doi: 10.1038/nrn3503 23686172 PMC3820118

[6] Meijer D, Veselič S, Calafiore C, Noppeney U. Integration of audiovisual spatial signals is not consistent with maximum likelihood estimation. Cortex. 2019 Oct;119:74–88. doi: 10.1016/j.cortex.2019.03.026 31082680 PMC6864592

[7] Rohe T, Noppeney U. Reliability-weighted integration of audiovisual signals can be modulated by top-down control. eNeuro. 2018;5(1):e0315–7. 10.1523/ENEURO.0315-17.2018 PMC5844059 29527567

[8] Vercillo T, Gori M. Attention to sound improves auditory reliability in audio-tactile spatial optimal integration. Front Integr Neurosci. 2015;9:34. doi: 10.3389/fnint.2015.00034 25999825 PMC4423351

[9] Odegaard B, Wozny DR, Shams L. The effects of selective and divided attention on sensory precision and integration. Neurosci Lett. 2016;614:24–8. doi: 10.1016/j.neulet.2015.12.039 26742638

[10] Badde S, Navarro KT, Landy MS. Modality-specific attention attenuates visual-tactile integration and recalibration effects by reducing prior expectations of a common source for vision and touch. Cognition. 2020;197:104170. doi: 10.1016/j.cognition.2019.104170 32036027 PMC7182122

[11] Rohe T, Noppeney U. Cortical Hierarchies Perform Bayesian Causal Inference in Multisensory Perception. PLoS Biol. 2015;13(2):e1002073. doi: 10.1371/journal.pbio.1002073 25710328 PMC4339735

[12] Rohe T, Noppeney U. Distinct computational principles govern multisensory integration in primary sensory and association cortices. Curr Biol. 2016;1(4):509–14. doi: 10.1016/j.cub.2015.12.056 26853368

[13] Aller M, Noppeney U. To integrate or not to integrate: Temporal dynamics of hierarchical Bayesian causal inference. PLoS Biol. 2019;17(4):e3000210. doi: 10.1371/journal.pbio.3000210 30939128 PMC6461295

[14] Macaluso E, Noppeney U, Talsma D, Vercillo T, Hartcher-O’Brien J, Adam R. The curious incident of attention in multisensory integration: Bottom-up vs. top-down. Multisens Res. 2016;29(6–7):557–83.

[15] Noppeney U. Perceptual Inference, Learning, and Attention in a Multisensory World. Annu Rev Neurosci. 2021;44(1):449–73. 33882258 10.1146/annurev-neuro-100120-085519

[16] Treue S. Neural correlates of attention in primate visual cortex. Trends Neurosci. 2001;24(5):295–300. doi: 10.1016/s0166-2236(00)01814-2 11311383

[17] Martinez-Trujillo JC, Treue S. Feature-based attention increases the selectivity of population responses in primate visual cortex. Curr Biol. 2004;14(9):744–51. doi: 10.1016/j.cub.2004.04.028 15120065

[18] Fischer J, Whitney D. Attention Narrows Position Tuning of Population Responses in V1. Curr Biol. 2009;19(16):1356–61. doi: 10.1016/j.cub.2009.06.059 19631540 PMC2757109

[19] Helbig HB, Ernst MO. Visual-haptic cue weighting is independent of modality-specific attention. J Vis. 2008;8(1):1–16. doi: 10.1167/8.1.21 18318624

[20] Körding KP, Beierholm U, Ma WJ, Quartz S, Tenenbaum JB, Shams L. Causal Inference in Multisensory Perception. PLoS ONE. 2007;2(9):e943. doi: 10.1371/journal.pone.0000943 17895984 PMC1978520

[21] Shams L, Beierholm UR. Causal inference in perception. Trends Cogn Sci. 2010;14(9):425–32. doi: 10.1016/j.tics.2010.07.001 20705502

[22] French RL, DeAngelis GC. Multisensory neural processing: from cue integration to causal inference. Curr Opin Physiol. 2020;16:8–13. doi: 10.1016/j.cophys.2020.04.004 32968701 PMC7505234

[23] Dokka K, Park H, Jansen M, DeAngelis GC, Angelaki DE. Causal inference accounts for heading perception in the presence of object motion. Proc Natl Acad Sci U S A. 2019;116(18):9060–5. doi: 10.1073/pnas.1820373116 30996126 PMC6500172

[24] Acerbi L, Dokka K, Angelaki DE, Ma WJ. Bayesian comparison of explicit and implicit causal inference strategies in multisensory heading perception. PLoS Comput Biol. 2018;14(7):e1006110. doi: 10.1371/journal.pcbi.1006110 30052625 PMC6063401

[25] Magnotti JF, Beauchamp MS. A Causal Inference Model Explains Perception of the McGurk Effect and Other Incongruent Audiovisual Speech. PLoS Comput Biol. 2017;13(2):e1005229. doi: 10.1371/journal.pcbi.1005229 28207734 PMC5312805

[26] Mohl JT, Pearson JM, Groh JM. Monkeys and humans implement causal inference to simultaneously localize auditory and visual stimuli. J Neurophysiol. 2020;124(3):715–27. doi: 10.1152/jn.00046.2020 32727263 PMC7509303

[27] Wozny DR, Beierholm UR, Shams L. Probability Matching as a Computational Strategy Used in Perception. PLoS Comput Biol. 2010;6(8):e1000871. doi: 10.1371/journal.pcbi.1000871 20700493 PMC2916852

[28] Rohe T, Ehlis A-C, Noppeney U. The neural dynamics of hierarchical Bayesian causal inference in multisensory perception. Nat Commun. 2019;10(1):1907. doi: 10.1038/s41467-019-09664-2 31015423 PMC6478901

[29] Cao Y, Summerfield C, Park H, Giordano BL, Kayser C. Causal inference in the multisensory brain. Neuron. 2019;102:1–12. doi: 10.1016/j.neuron.2019.03.019 31047778

[30] Shapleske J, Rossell S, Woodruff PW, David A. The planum temporale: a systematic, quantitative review of its structural, functional and clinical significance. Brain Res Rev. 1999;29(1):26–49. doi: 10.1016/s0165-0173(98)00047-2 9974150

[31] Battal C, Rezk M, Mattioni S, Vadlamudi J, Collignon O. Representation of auditory motion directions and sound source locations in the human planum temporale. J Neurosci. 2019;39(12):2208–20. doi: 10.1523/JNEUROSCI.2289-18.2018 30651333 PMC6433766

[32] Barrett DJK, Hall DA. Response preferences for “what” and “where” in human non-primary auditory cortex. Neuroimage. 2006;32(2):968–77. doi: 10.1016/j.neuroimage.2006.03.050 16733092

[33] Ahveninen J, Kopčo N, Jääskeläinen IP. Psychophysics and neuronal bases of sound localization in humans. Hear Res. 2014;307(2):86–97. doi: 10.1016/j.heares.2013.07.008 23886698 PMC3858499

[34] Jones SA, Beierholm U, Meijer D, Noppeney U. Older adults sacrifice response speed to preserve multisensory integration performance. Neurobiol Aging. 2019;84:148–57. doi: 10.1016/j.neurobiolaging.2019.08.017 31586863

[35] Noppeney U, Ostwald D, Werner S. Perceptual decisions formed by accumulation of audiovisual evidence in prefrontal cortex. J Neurosci. 2010;30(21):7434–46. doi: 10.1523/JNEUROSCI.0455-10.2010 20505110 PMC6632395

[36] Werner S, Noppeney U. Distinct functional contributions of primary sensory and association areas to audiovisual integration in object categorization. J Neurosci. 2010;30(7):2662–75. doi: 10.1523/JNEUROSCI.5091-09.2010 20164350 PMC6634553

[37] Carrasco M. Visual attention: The past 25 years. Vision Res. 2011;51(13):1484–525. doi: 10.1016/j.visres.2011.04.012 21549742 PMC3390154

[38] Posner MI, Snyder CR, Davidson BJ. Attention and the detection of signals. J Exp Psychol Gen. 1980;109(2):160–74. 7381367

[39] Spence C, Nicholls ME, Driver J. The cost of expecting events in the wrong sensory modality. Percept Psychophys. 2001;63(2):330–6. doi: 10.3758/bf03194473 11281107

[40] Giessing C, Thiel CM, Stephan KE, Rösler F, Fink GR. Visuospatial attention: How to measure effects of infrequent, unattended events in a blocked stimulus design. Neuroimage. 2004;23(4):1370–81. doi: 10.1016/j.neuroimage.2004.08.008 15589101

[41] Natale E, Marzi CA, Macaluso E. Right temporal-parietal junction engagement during spatial reorienting does not depend on strategic attention control. Neuropsychologia. 2010;48(4):1160–4. doi: 10.1016/j.neuropsychologia.2009.11.012 19932706

[42] Corbetta M, Shulman GL. Control of goal-directed and stimulus-driven attention in the brain. Nat Rev Neurosci. 2002;3(3):201–15. doi: 10.1038/nrn755 11994752

[43] Corbetta M, Patel G, Shulman GL. The reorienting system of the human brain: from environment to theory of mind. Neuron. 2008;58(3):306–24. doi: 10.1016/j.neuron.2008.04.017 18466742 PMC2441869

[44] Santangelo V, Fagioli S, Macaluso E. The costs of monitoring simultaneously two sensory modalities decrease when dividing attention in space. Neuroimage. 2010;49(3):2717–27. doi: 10.1016/j.neuroimage.2009.10.061 19878728

[45] Shomstein S, Yantis S. Control of attention shifts between vision and audition in human cortex. J Neurosci. 2004;24(47):10702–6. doi: 10.1523/JNEUROSCI.2939-04.2004 15564587 PMC6730120

[46] Geng JJ. Attentional Mechanisms of Distractor Suppression. Curr Dir Psychol Sci. 2014;23(2):147–53.

[47] Rohe T, Noppeney U. Sensory reliability shapes Bayesian Causal Inference in perception via two mechanisms. J Vis. 2015;15(5):1–16. doi: 10.1167/15.5.1 26067540

[48] Atilgan H, Town SM, Wood KC, Jones GP, Maddox RK, Lee AKC, et al. Integration of visual information in auditory cortex promotes auditory scene analysis through multisensory binding. Neuron. 2018;97(3):640, e4–55. doi: 10.1016/j.neuron.2017.12.034 29395914 PMC5814679

[49] Bizley JK, Nodal FR, Bajo VM, Nelken I, King AJ. Physiological and anatomical evidence for multisensory interactions in auditory cortex. Cereb Cortex. 2007;17(9):2172–89. doi: 10.1093/cercor/bhl128 17135481 PMC7116518

[50] Fiebelkorn IC, Foxe JJ, Molholm S. Dual mechanisms for the cross-sensory spread of attention: How much do learned associations matter? Cereb Cortex. 2010;20(1):109–20. doi: 10.1093/cercor/bhp083 19395527 PMC2792190

[51] Ghazanfar AA, Schroeder CE. Is neocortex essentially multisensory? Trends Cogn Sci. 2006;10(6):278–85. doi: 10.1016/j.tics.2006.04.008 16713325

[52] Kayser C, Logothetis NK. Do early sensory cortices integrate cross-modal information? Brain Struct Funct. 2007;212(2):121–32. doi: 10.1007/s00429-007-0154-0 17717687

[53] Kayser C, Petkov CI, Logothetis NK. Visual modulation of neurons in auditory cortex. Cereb Cortex. 2008;18(7):1560–74. doi: 10.1093/cercor/bhm187 18180245

[54] Lakatos P, Chen CM, O’Connell MN, Mills A, Schroeder CE. Neuronal oscillations and multisensory interaction in primary auditory cortex. Neuron. 2007;53(2):279–92. doi: 10.1016/j.neuron.2006.12.011 17224408 PMC3717319

[55] Martuzzi R, Murray MM, Michel CM, Thiran JP, Maeder PP, Clarke S, et al. Multisensory interactions within human primary cortices revealed by BOLD dynamics. Cereb Cortex. 2007;17(7):1672–9. doi: 10.1093/cercor/bhl077 16968869

[56] Noesselt T, Rieger JW, Schoenfeld MA, Kanowski M, Hinrichs H, Heinze H-J, et al. Audiovisual temporal correspondence modulates human multisensory superior temporal sulcus plus primary sensory cortices. J Neurosci. 2007;27(42):11431–41. doi: 10.1523/JNEUROSCI.2252-07.2007 17942738 PMC2957075

[57] Werner S, Noppeney U. Superadditive responses in superior temporal sulcus predict audiovisual benefits in object categorization. Cereb Cortex. 2010;20(8):1829–42. doi: 10.1093/cercor/bhp248 19923200

[58] Driver J, Noesselt T. Multisensory interplay reveals crossmodal influences on “sensory-specific” brain regions, neural responses, and judgments. Neuron. 2008;57(1):11–23. doi: 10.1016/j.neuron.2007.12.013 18184561 PMC2427054

[59] Ortiz-Rios M, Azevedo FAC, Kusmierek P, Balla DZ, Munk MH, Keliris GA, et al. Widespread and opponent fMRI signals represent sound location in macaque auditory cortex. Neuron. 2017;93(4):971–83. doi: 10.1016/j.neuron.2017.01.013 28190642 PMC5757378

[60] Aller M, Mihalik A, Noppeney U. Audiovisual adaptation is expressed in spatial and decisional codes. bioRxiv. 2021. 10.1038/s41467-022-31549-0 PMC9262908 35798733

[61] Mihalik A, Noppeney U. Causal inference in audiovisual perception. J Neurosci. 2020;40(34):6600–12. doi: 10.1523/JNEUROSCI.0051-20.2020 32669354 PMC7486655

[62] Gau R, Noppeney U. How prior expectations shape multisensory perception. Neuroimage. 2016;124:876–86. doi: 10.1016/j.neuroimage.2015.09.045 26419391

[63] Bertelson P, Vroomen J, De Gelder B, Driver J. The ventriloquist effect does not depend on the direction of deliberate visual attention. Percept Psychophys. 2000;62(2):321–32. doi: 10.3758/bf03205552 10723211

[64] Vroomen J, Bertelson P, de Gelder B. The ventriloquist effect does not depend on the direction of automatic visual attention. Percept Psychophys. 2001;63(4):651–9. doi: 10.3758/bf03194427 11436735

[65] Hou H, Zheng Q, Zhao Y, Pouget A, Gu Y. Neural Correlates of Optimal Multisensory Decision Making under Time-Varying Reliabilities with an Invariant Linear Probabilistic Population Code. Neuron. 2019;104(5):1010–21. doi: 10.1016/j.neuron.2019.08.038 31607423

[66] Talsma D, Doty TJ, Woldorff MG. Selective attention and audiovisual integration: is attending to both modalities a prerequisite for early integration? Cereb Cortex. 2007;17(3):679–90. doi: 10.1093/cercor/bhk016 16707740

[67] Faul F, Erdfelder E, Buchner A, Lang A-G. Statistical power analyses using G*Power 3.1: Tests for correlation and regression analyses. Behav Res Methods. 2009;41(4):1149–60. doi: 10.3758/BRM.41.4.1149 19897823

[68] Oldfield RC. The assessment and analysis of handedness: the Edinburgh inventory. Neuropsychologia. 1971;9(1):97–113. doi: 10.1016/0028-3932(71)90067-4 5146491

[69] Blignaut P. Fixation identification: The optimum threshold for a dispersion algorithm. Atten Percept Psychophys. 2009;71(4):881–95. doi: 10.3758/APP.71.4.881 19429966

[70] Gardner WG, Martin KD. HRTF measurements of a KEMAR. J Acoust Soc Am. 1995;97(6):3907–8.

[71] Zuanazzi A, Noppeney U. Additive and interactive effects of spatial attention and expectation on perceptual decisions. Sci Rep. 2018;8(1):6732. doi: 10.1038/s41598-018-24703-6 29712941 PMC5928039

[72] Zuanazzi A, Noppeney U. Distinct Neural Mechanisms of Spatial Attention and Expectation Guide Perceptual Inference in a Multisensory World. J Neurosci. 2019;39(12):2301–12. doi: 10.1523/JNEUROSCI.2873-18.2019 30659086 PMC6433765

[73] Zuanazzi A, Noppeney U. The intricate interplay of spatial attention and expectation: A multisensory perspective. Multisens Res. 2020;33(4–5):383–416. doi: 10.1163/22134808-20201482 31940592

[74] Zuanazzi A, Noppeney U. Modality-specific and multisensory mechanisms of spatial attention and expectation. J Vis. 2020;20(8):1–16. doi: 10.1167/jov.20.8.1 32744617 PMC7438668

[75] Kleiner M, Brainard DH, Pelli DG. What’s new in Psychtoobox-3? Perception. 2007;36:14.

[76] Nagelkerke NJD. A note on a general definition of the coefficient of determination. Biometrika. 1991;78(3):691–2.

[77] Kass RE, Raftery AE. Bayes factors. J Am Stat Assoc. 1995 Jun;90(430):773–95.

[78] Rigoux L, Stephan KE, Friston KJ, Daunizeau J. Bayesian model selection for group studies—Revisited. Neuroimage. 2014;84:971–85. doi: 10.1016/j.neuroimage.2013.08.065 24018303

[79] Friston KJ, Holmes AP, Worsley KJ, Poline J-P, Frith CD, Frackowiak RSJ. Statistical parametric maps in functional imaging: A general linear approach. Hum Brain Mapp. 1994;2(4):189–210.

[80] Hebart MN, Görgen K, Haynes J-D, Dubois J. The Decoding Toolbox (TDT): a versatile software package for multivariate analyses of functional imaging data. Front Neuroinform. 2015;8:1–18. doi: 10.3389/fninf.2014.00088 25610393 PMC4285115

[81] Chang C-C, Lin C-J. LIBSVM: A library for support vector machines. ACM Trans Intell Syst Technol. 2011;2(3):1–27.

[82] Wang L, Mruczek REB, Arcaro MJ, Kastner S. Probabilistic maps of visual topography in human cortex. Cereb Cortex. 2015;25(10):3911–31. doi: 10.1093/cercor/bhu277 25452571 PMC4585523

[83] Rauschecker JP, Tian B. Mechanisms and streams for processing of “what” and “where” in auditory cortex. Proc Natl Acad Sci U S A. 2000;97(22):11800–6. doi: 10.1073/pnas.97.22.11800 11050212 PMC34352

[84] Eickhoff SB, Stephan KE, Mohlberg H, Grefkes C, Fink GR, Amunts K, et al. A new SPM toolbox for combining probabilistic cytoarchitectonic maps and functional imaging data. Neuroimage. 2005;25(4):1325–35. doi: 10.1016/j.neuroimage.2004.12.034 15850749

[85] Destrieux C, Fischl B, Dale A, Halgren E. Automatic parcellation of human cortical gyri and sulci using standard anatomical nomenclature. Neuroimage. 2010;53(1):1–15. doi: 10.1016/j.neuroimage.2010.06.010 20547229 PMC2937159

