# Supplementary material for: Attention controls multisensory perception via two distinct mechanisms at different levels of the cortical hierarchy
Source: PLoS Biol. 2021 Nov 18;19(11):e3001465. doi: 10.1371/journal.pbio.3001465 (PMC8639080; doi:10.1371/journal.pbio.3001465)
Supplement: S1 Text — Unisensory auditory localisation inside the scanner. (DOCX) [file pbio.3001465.s004.docx]

## S1 Text. Unisensory auditory localisation inside the scanner.

## Methods

Prior to the main experiment, we performed unisensory auditory localisation inside the scanner to ensure that observers were able to locate sounds in the scanner noise and that those elicited lateralized activations in the posterior superior temporal gyrus (specifically, planum temporale) for auditory space perception[1–4].

**Experimental design and procedure.** We used the same auditory stimuli as in the audiovisual ventriloquist paradigm of the main experiment. Signals were sampled from three positions along the azimuth (-9°, 0° or 9° visual angle). To increase design efficiency, auditory locations were pseudo-randomized across trials. In each trial, a 750 ms inter-trial interval was followed by a 50 ms auditory signal (in one of the three azimuthal positions) and a 2 seconds response interval, during which participants reported as accurately as possible their perceived auditory location using a keypad (S1a Figure). Throughout the task, participants fixated a cross (1° diameter) in the centre of the screen. They completed 2 scanning runs (3 conditions × 42 trails / condition / run × 2 runs = 252 trails in total), with each run divided into 7 task blocks (18 trials / block) and 7 fixation blocks presented in an interleaved fashion. Participants were familiarized with stimuli and procedure via one practice run before entering the scanner room.

**Experimental setup.** The experiment was presented via Psychtoolbox version 3.0.11[5] running under MATLAB R2011b (MathWorks Inc.) on a MacBook Pro (Mac OSX 10.6.8). Auditory stimuli were played using MR-compatible headphones (MR Confon HP-VS03). Participants gave responses via one MR-compatible keypad (NATA LXPAD 1×5-10M, NATAtech.com) with their right hand.

**MRI data acquisition.** We acquired T2*-weighted axial echoplanar images (EPI) with blood-oxygenation-level-dependent contrast (gradient echo, SENSE factor of 2, TR = 2800 ms, TE = 40 ms, flip angle = 90°, FOV = 192 × 192 × 114 mm^2^, 38 axial slices acquired in sequential ascending direction, voxel size = 2.5 × 2.5 × 2.5 mm^3^ + 0.5 mm interslice gap). A total of 128 volumes times 2 runs were acquired, after discarding the first four volumes of each run to allow for T1 equilibration effects. Data acquisition was performed during one scanning day.

**Behavioural data analysis.** Participants’ spatial localization reliability was quantified by computing the root-mean-square error (RMSE) between participants’ reported location and signal’s true location, combining all spatial locations. Participants were considered outliers and hence excluded if their RMSE exceeded 5.5° (thresholds defined as two standards deviations above the group mean in a preliminary pilot study).

**fMRI data analysis.** MRI data were analyzed using SPM12 (Wellcome Department of Imaging Neuroscience, London; www.fil.ion.ucl.ac.uk/spm[6]). Scans from each subject were realigned using the first as a reference, unwarped, slice-time corrected and spatially normalized into MNI standard space using parameters from segmentation of the T1 structural image[7], resampled to a spatial resolution of 2 × 2 × 2 mm^3^ and spatially smoothed with a Gaussian kernel of 8 mm full-width at half-maximum. The time series of all voxels were high-pass filtered to 1/128 Hz. In an event-related design, unit impulses representing stimuli onsets were convolved with a canonical hemodynamic response function and its first temporal derivative. The three experimental conditions (i.e. three auditory locations) were included as regressors in the design matrix. Realignment parameters were also added as nuisance covariates to account for noise due to residual head motion artefacts. The voxel-wise magnitude of the BOLD signal in response to the audiovisual onsets was defined by the parameter estimates pertaining to the canonical hemodynamic response function. Subject-specific contrast images (each experimental condition versus baseline summed over the two runs) were passed to a second-level ANOVA, where contrasts of interest were defined. Inferences were made at the second (i.e. between-subjects or random effects) level[6], where we tested for the effect of unisensory auditory localisation collapsing across spatial locations (Task > Baseline) and separately for left versus right lateralised sounds (SoundL > SoundR; SoundR > SoundL). Whole-brain activations are reported at p < 0.05 (Family-Wise Error corrected) at the peak level[8] in S11 Table.

## Results

Behavioural results. Every participant showed RMSE < 5.5° (i.e. no participant was excluded), resulting in group mean RMSE (± SEM) = 3.27° (± 0.29°). In addition, participants’ reported spatial locations were strongly correlated with the true auditory signal locations (group mean Pearson's correlation coefficient r [95% CI] = 0.92 [0.89, 0.94], p < 0.001 for one-tailed permutation test on r > 0; individual correlation coefficients were Fisher-z transformed prior to statistical testing, and the group mean was inverse-transformed).These results demonstrate that participants were able to locate sounds accurately even when scanner noise was present.

fMRI results. In line with our predictions[1–4], auditory localisation (i.e. Task > Baseline) increased activations in bilateral planum temporale; in addition, we found increased activations in bilateral superior frontal gyrus, in right parietal operculum and in a motor network encompassing the left pre- and post-central sulcus and the right cerebellum (mapping the hand area), which reflected motor response execution (S11 Table).

Notably, lateralised sounds activated contralateral planum temporale (S1b Figure), in accordance with emerging theories of opponent channel coding both in humans[9] and non-human primates[10]. Overall, behavioural and fMRI data provide converging evidence that participants successfully processed the auditory information for spatial localization in the scanning environment.

## References

1. Shapleske J, Rossell S., Woodruff PW., David A. The planum temporale: a systematic, quantitative review of its structural, functional and clinical significance. Brain Res Rev. 1999;29(1):26–49.

2. Battal C, Rezk M, Mattioni S, Vadlamudi J, Collignon O. Representation of auditory motion directions and sound source locations in the human planum temporale. J Neurosci. 2019;39(12):2208–20.

3. Barrett DJK, Hall DA. Response preferences for “what” and “where” in human non-primary auditory cortex. Neuroimage. 2006;32(2):968–77.

4. Ahveninen J, Kopčo N, Jääskeläinen IP. Psychophysics and neuronal bases of sound localization in humans. Hear Res. 2014;307(2):86–97.

5. Kleiner M, Brainard DH, Pelli DG. What’s new in Psychtoobox-3? Perception. 2007;36:14.

6. Friston KJ, Holmes AP, Worsley KJ, Poline J-P, Frith CD, Frackowiak RSJ. Statistical parametric maps in functional imaging: A general linear approach. Hum Brain Mapp. 1994;2(4):189–210.

7. Ashburner J, Friston KJ. Unified segmentation. Neuroimage. 2005;26(3):839–51.

8. Friston KJ, Worsley KJ, Frackowiak RSJ, Mazziotta JC, Evans AC. Assessing the significance of focal activations using their spatial extent. Hum Brain Mapp. 1994;1(3):210–20.

9. Derey K, Valente G, De Gelder B, Formisano E. Opponent coding of sound location (azimuth) in planum temporale is robust to sound-level variations. Cereb Cortex. 2016;26(1):450–64.

10. Ortiz-Rios M, Azevedo FAC, Kusmierek P, Balla DZ, Munk MH, Keliris GA, et al. Widespread and opponent fMRI signals represent sound location in macaque auditory cortex. Neuron. 2017;93(4):971–83.
