# Supplementary material for: Attention controls multisensory perception via two distinct mechanisms at different levels of the cortical hierarchy
Source: PLoS Biol. 2021 Nov 18;19(11):e3001465. doi: 10.1371/journal.pbio.3001465 (PMC8639080; doi:10.1371/journal.pbio.3001465)
Supplement: S1 Table — Across participants’ mean (±SEM) as a function of prestimulus attention (attA, auditory; attV, visual), poststimulus report (repA: auditory; repV: visual), and audiovisual spatial disparity (dispN: no disparity; dispL: low; dispH: high). (DOCX) [file pbio.3001465.s005.docx]

**S1 Table. Response times (RT) in the psychophysics and fMRI experiments.**

| **RT (ms)** | attArepA | attVrepA | attArepV | attVrepV |
| --- | --- | --- | --- | --- |
| **Psychophysics** |  |  |  |  |
| dispN | 813.58 (±29.47) | 1090.31 (±31.57) | 924.47 (±29.71) | 672.74 (±25.70) |
| dispL | 886.23 (±29.25) | 1158.21 (±31.74) | 989.13 (±30.27) | 673.38 (±23.50) |
| dispH | 980.78 (±32.30) | 1196.17 (±33.81) | 1034.61 (±36.37) | 714.72 (±23.02) |
| **fMRI** |  |  |  |  |
| dispN | 699.31 (±43.99) | 886.36 (±43.67) | 774.94 (±44.10) | 574.80 (±36.62) |
| dispL | 810.20 (±47.28) | 973.04 (±52.79) | 812.41 (±48.05) | 589.13 (±36.04) |
| dispH | 821.97 (±36.73) | 972.20 (±48.26) | 833.90 (±43.75) | 636.26 (±41.78) |

Across participants' mean (±SEM) as a function of pre-stimulus attention (attA: auditory; attV: visual), post-stimulus report (repA: auditory; repV: visual) and audiovisual spatial disparity (dispN: no disparity; dispL: low; dispH: high).
