# Supplementary material for: Attention controls multisensory perception via two distinct mechanisms at different levels of the cortical hierarchy
Source: PLoS Biol. 2021 Nov 18;19(11):e3001465. doi: 10.1371/journal.pbio.3001465 (PMC8639080; doi:10.1371/journal.pbio.3001465)
Supplement: S2 Table — Main effects and interactions of the 2 (prestimulus attention, Att: attA, attV) × 2 (poststimulus report, Rep: repA, repV) × 3 (audiovisual spatial disparity, Disp: low, high) repeated measures ANOVA. Greenhouse–Geisser correction is applied to degrees of freedom (df1 and df2) in case of violation of sphericity (Mauchly test). (DOCX) [file pbio.3001465.s006.docx]

**S2 Table. Statistical results of response times (RT) in the psychophysics and fMRI experiments.**

| **RT** | Att | Rep | Disp | Att×Rep | Att×Disp | Rep×Disp | Att×Rep×Disp |
| --- | --- | --- | --- | --- | --- | --- | --- |
| **Psychophysics** |  |  |  |  |  |  |  |
| F-value (df1, df2) | 3.75 (1, 26) | 86.00 (1, 26) | 67.19 (2, 52) | 247.33 (1, 26) | 7.64 (1.51, 39.15) | 9.44 (1.40, 36.50) | 3.57 (1.41, 36.78) |
| Two-tailed p-value | .064 | **.000** | **.000** | **.000** | **.004** | **.002** | .053 |
| Effect size (ηp^2^) | .13 | .77 | .72 | .90 | .23 | .27 | .12 |
| **fMRI** |  |  |  |  |  |  |  |
| F-value (df1, df2) | 3.28 (1, 11) | 27.95 (1, 11) | 59.21 (2, 22) | 128.59 (1, 11) | 2.02 (2, 22) | 31.29 (2, 22) | 1.67 (2, 22) |
| Two-tailed p-value | .098 | **.000** | **.000** | **.000** | .157 | .**000** | .211 |
| Effect size (ηp^2^) | .23 | .72 | .84 | .92 | .15 | .74 | .13 |

Main effects and interactions of the 2 (pre-stimulus attention, Att: attA, attV) × 2 (post-stimulus report, Rep: repA, repV) × 3 (audiovisual spatial disparity, Disp: low, high) repeated measures ANOVA. Greenhouse-Geisser correction is applied to degrees of freedom (df1, df2) in case of violation of sphericity (Mauchly's test).
