# Supplementary material for: Attention controls multisensory perception via two distinct mechanisms at different levels of the cortical hierarchy
Source: PLoS Biol. 2021 Nov 18;19(11):e3001465. doi: 10.1371/journal.pbio.3001465 (PMC8639080; doi:10.1371/journal.pbio.3001465)
Supplement: S3 Table — Effect of audiovisual spatial incongruency [AVincongruent (AV disparity ≠ 0°) > AVcongruent (AV disparity = 0°)] and congruency [AVcongruent (AV disparity = 0°) > AVincongruent (AV disparity ≠ 0°)]. p-Values are FWE corrected at the peak level for multiple comparisons within the entire brain. FWE, family-wise error; L, left; R, right. (DOCX) [file pbio.3001465.s007.docx]

**S3 Table. fMRI univariate results: audiovisual spatial (in)congruency.**

| Brain regions | MNI coordinates (mm) | | | Cluster size (voxels) | z-score (peak) | p _FWE-corrected_ (peak) |
| --- | --- | --- | --- | --- | --- | --- |
|  | x | y | z |  |  |  |
| AVincongruent > AVcongruent |  |  |  |  |  |  |
| R superior frontal gyrus | 22 | 0 | 52 | 574 | > 8 | 0.000 |
| L superior frontal gyrus | -28 | -6 | 60 | 512 | 7.57 | 0.000 |
| L superior frontal gyrus | -2 | 14 | 48 | 778 | > 8 | 0.000 |
| R anterior cingulate gyrus | 10 | 20 | 38 |  | 7.12 | 0.000 |
| R anterior insula | 34 | 20 | 4 | 274 | 7.22 | 0.000 |
| L anterior insula | -30 | 24 | 0 | 194 | 6.72 | 0.000 |
| R superior parietal lobule | 16 | -70 | 54 | 138 | 6.24 | 0.000 |
| L superior parietal lobule | -16 | -70 | 52 | 70 | 5.73 | 0.000 |
| R inferior frontal gyrus (pars opercularis) | 44 | 6 | 28 | 380 | 6.06 | 0.000 |
| L inferior frontal gyrus (pars opercularis) | -54 | 4 | 20 | 44 | 5.63 | 0.001 |
| R intraparietal sulcus | 34 | -44 | 44 | 68 | 5.51 | 0.001 |
| L intraparietal sulcus | -42 | -36 | 42 | 65 | 5.31 | 0.003 |
|  |  |  |  |  |  |  |
| AVcongruent > AVincongruent |  |  |  |  |  |  |
| L angular gyrus | -52 | -68 | 26 | 10 | 4.95 | 0.014 |
| R frontopolar gyrus | 6 | 62 | -6 | 7 | 4.85 | 0.022 |
|  |  |  |  |  |  |  |

Effect of audiovisual spatial incongruency [AVincongruent (AV disparity ≠ 0°) > AVcongruent (AV disparity = 0°)] and congruency [AVcongruent (AV disparity = 0°) > AVincongruent (AV disparity ≠ 0°)]. p-values are FWE-corrected at the peak level for multiple comparisons within the entire brain. L: left; R: right.
