# Supplementary material for: Attention controls multisensory perception via two distinct mechanisms at different levels of the cortical hierarchy
Source: PLoS Biol. 2021 Nov 18;19(11):e3001465. doi: 10.1371/journal.pbio.3001465 (PMC8639080; doi:10.1371/journal.pbio.3001465)
Supplement: S4 Table — Effect of cue invalidity [Invalid (attVrepA & attArepV) > Valid (attArepA & attVrepV)] and validity [Valid (attArepA & attVrepV) > Invalid (attVrepA & attArepV)], where attA: auditory prestimulus attention; attV: visual prestimulus attention; repA: auditory poststimulus report; repV: visual poststimulus report. p-Values are FWE corrected at the peak level for multiple comparisons within the entire brain. FWE, family-wise error; L, left; R, right. (DOCX) [file pbio.3001465.s008.docx]

**S4 Table. fMRI univariate results: cue (in)validity.**

| Brain regions | MNI coordinates (mm) | | | Cluster size (voxels) | z-score (peak) | p _FWE-corrected_ (peak) |
| --- | --- | --- | --- | --- | --- | --- |
|  | x | y | z |  |  |  |
| Invalid > Valid |  |  |  |  |  |  |
| L superior frontal gyrus | -4 | 8 | 52 | 7113 | > 8 | 0.000 |
| L superior frontal gyrus | -24 | -6 | 56 |  | > 8 | 0.000 |
| R superior frontal gyrus | 24 | -4 | 52 |  | > 8 | 0.000 |
| L inferior frontal gyrus (pars opercularis) | -46 | 2 | 32 |  | > 8 | 0.000 |
| R anterior cingulate gyrus | 10 | 18 | 36 |  | 7.41 | 0.000 |
| L anterior cingulate gyrus | -10 | 18 | 32 |  | 5.11 | 0.007 |
| L intraparietal sulcus | -28 | -54 | 46 | 4129 | > 8 | 0.000 |
| L precuneus | -6 | -62 | 48 |  | > 8 | 0.000 |
| L superior parietal lobule | -14 | -68 | 50 |  | > 8 | 0.000 |
| R intraparietal sulcus | 34 | -42 | 44 | 43 | 5.40 | 0.002 |
| L middle frontal gyrus | -28 | 48 | 12 | 472 | > 8 | 0.000 |
| R middle frontal gyrus | 32 | 44 | 26 | 109 | 5.93 | 0.000 |
| R fusiform gyrus | 34 | -54 | -20 | 635 | > 8 | 0.000 |
| R cerebellum | 38 | -54 | -32 |  | 6.34 | 0.000 |
| L fusiform gyrus | -32 | -50 | -18 | 613 | 7.77 | 0.000 |
| L cerebellum | -30 | -56 | -32 |  | 6.40 | 0.000 |
| R calcarine cortex | 8 | -76 | 8 | 771 | 6.17 | 0.000 |
| L calcarine cortex | -10 | -80 | 6 |  | 5.85 | 0.000 |
|  |  |  |  |  |  |  |
| Valid > Invalid |  |  |  |  |  |  |
| R inferior frontal gyrus (pars triangularis) | 46 | 40 | 4 | 36 | 5.23 | 0.004 |
| R lateral orbital gyrus | 34 | 38 | -10 | 13 | 5.00 | 0.011 |
| L lateral orbital gyrus | -34 | -36 | -12 | 7 | 4.80 | 0.028 |
|  |  |  |  |  |  |  |

Effect of cue invalidity [Invalid (attVrepA & attArepV) > Valid (attArepA & attVrepV)] and validity [Valid (attArepA & attVrepV) > Invalid (attVrepA & attArepV)], where attA: auditory pre-stimulus attention; attV: visual pre-stimulus attention; repA: auditory post-stimulus report; repV: visual post-stimulus report. p-values are FWE-corrected at the peak level for multiple comparisons within the entire brain. L: left; R: right.
