# Supplementary material for: Attention controls multisensory perception via two distinct mechanisms at different levels of the cortical hierarchy
Source: PLoS Biol. 2021 Nov 18;19(11):e3001465. doi: 10.1371/journal.pbio.3001465 (PMC8639080; doi:10.1371/journal.pbio.3001465)
Supplement: S5 Table — Effect of cue invalidity separately for auditory (attVrepA > attArepA) and visual report (attArepV > attVrepV), where attA: auditory attention; attV: visual attention; repA: auditory report; repV: visual report. p-Values are FWE corrected at the peak level for multiple comparisons within the entire brain. FWE, family-wise error; L, left; R, right. (DOCX) [file pbio.3001465.s009.docx]

**S5 Table. fMRI univariate results: cue invalidity separately for auditory and visual report.**

| Brain regions | MNI coordinates (mm) | | | Cluster size (voxels) | z-score (peak) | p _FWE-corrected_ (peak) |
| --- | --- | --- | --- | --- | --- | --- |
|  | x | y | z |  |  |  |
| Invalid > Valid (repA) |  |  |  |  |  |  |
| L superior frontal gyrus | -22 | -4 | 58 | 722 | > 8 | 0.000 |
| R superior frontal gyrus | 24 | -4 | 48 | 173 | 5.72 | 0.000 |
| L superior frontal gyrus | -4 | 12 | 48 | 423 | 7.65 | 0.000 |
| L superior parietal lobule | -20 | -66 | 46 | 573 | 6.26 | 0.000 |
| L precuneus | -6 | -64 | 50 |  | 5.75 | 0.000 |
| L intraparietal sulcus | -28 | -54 | 46 |  | 5.73 | 0.000 |
| R superior parietal lobule | 24 | -64 | 44 | 3 | 4.77 | 0.031 |
| L middle frontal gyrus | -28 | 50 | 10 | 103 | 5.66 | 0.000 |
| L supramarginal gyrus | -42 | -36 | 46 | 56 | 5.48 | 0.001 |
| R fusiform gyrus | 32 | -52 | -20 | 27 | 5.42 | 0.001 |
| L fusiform gyrus | -32 | -50 | -20 | 12 | 4.91 | 0.017 |
|  |  |  |  |  |  |  |
| Invalid > Valid (repV) |  |  |  |  |  |  |
| L superior frontal gyrus | -4 | 8 | 52 | 1647 | > 8 | 0.000 |
| L superior frontal gyrus | -28 | -6 | 58 |  | > 8 | 0.000 |
| R superior frontal gyrus | 30 | -6 | 52 | 495 | 6.32 | 0.000 |
| L intraparietal sulcus | -32 | -50 | 46 | 884 | 6.85 | 0.000 |
| L supramarginal gyrus | -48 | -36 | 48 |  | 5.78 | 0.000 |
| L precuneus | -6 | -58 | 46 | 268 | 6.67 | 0.000 |
| R precuneus | 6 | -60 | 48 |  | 5.82 | 0.000 |
| L middle frontal gyrus | -28 | 46 | 14 | 40 | 5.41 | 0.002 |
| L inferior frontal gyrus (pars opercularis) | -46 | 2 | 32 | 705 | 7.29 | 0.000 |
|  |  |  |  |  |  |  |

Effect of cue invalidity separately for auditory (attVrepA > attArepA) and visual report (attArepV > attVrepV), where attA: auditory attention; attV: visual attention; repA: auditory report; repV: visual report. p-values are FWE-corrected at the peak level for multiple comparisons within the entire brain. L: left; R: right.
