# Supplementary material for: Attention controls multisensory perception via two distinct mechanisms at different levels of the cortical hierarchy
Source: PLoS Biol. 2021 Nov 18;19(11):e3001465. doi: 10.1371/journal.pbio.3001465 (PMC8639080; doi:10.1371/journal.pbio.3001465)
Supplement: S6 Table — Effect of auditory relative to visual report (repA > repV) and vice versa (repV > repA); effect of auditory relative to visual attention (attA > attV) and vice versa (attV > attA). p-Values are FWE corrected at the peak level for multiple comparisons within the entire brain. FWE, family-wise error; L, left; R, right. (DOCX) [file pbio.3001465.s010.docx]

**S6 Table. fMRI univariate results: post-stimulus report and pre-stimulus attention.**

| Brain regions | MNI coordinates (mm) | | | Cluster size (voxels) | z-score (peak) | p _FWE-corrected_ (peak) |
| --- | --- | --- | --- | --- | --- | --- |
|  | x | y | z |  |  |  |
| repA > repV |  |  |  |  |  |  |
| R anterior insula | 34 | 22 | 0 | 1927 | > 8 | 0.000 |
| R inferior precentral sulcus | 44 | 8 | 26 |  | 7.14 | 0.000 |
| R inferior frontal gyrus (pars opercularis) | 52 | 20 | 28 |  | 6.93 | 0.000 |
| L anterior insula | -34 | 20 | 0 | 680 | > 8 | 0.000 |
| L superior frontal gyrus | -1 | 20 | 46 | 1200 | > 8 | 0.000 |
| R superior frontal gyrus | 8 | 22 | 38 |  | > 8 | 0.000 |
| R anterior cingulate gyrus | 8 | 22 | 38 |  | > 8 | 0.000 |
| L inferior frontal gyrus (pars triangularis) | -40 | 26 | 22 | 1306 | > 8 | 0.000 |
| L inferior precentral sulcus | -50 | 8 | 26 |  | > 8 | 0.000 |
| L superior frontal sulcus | -24 | -4 | 58 | 156 | 5.99 | 0.000 |
| R superior frontal sulcus | 24 | 2 | 54 | 2 | 4.74 | 0.036 |
| L intraparietal sulcus | -32 | -48 | 38 | 49 | 5.33 | 0.002 |
| R intraparietal sulcus | 38 | -44 | 44 | 30 | 5.23 | 0.004 |
| repV > repA |  |  |  |  |  |  |
| L posterior cingulate gyrus | -4 | -38 | 42 | 2377 | > 8 | 0.000 |
| L precuneus | -6 | -54 | 18 |  | 5.10 | 0.000 |
| L angular gyrus | -54 | -56 | 28 | 1531 | > 8 | 0.000 |
| L middle occipital gyrus | -30 | -72 | 24 |  | 5.46 | 0.001 |
| R angular gyrus | 52 | -50 | 32 | 806 | > 8 | 0.000 |
| R frontopolar gyrus | 6 | 62 | -6 | 1876 | 7.21 | 0.000 |
| L frontopolar gyrus | -12 | 62 | 12 |  | 6.70 | 0.000 |
| L middle frontal gyrus | -32 | 20 | 42 | 1184 | 7.17 | 0.000 |
| R middle frontal gyrus | 36 | 28 | 42 | 345 | 5.89 | 0.000 |
| L postcentral gyrus | -40 | -26 | 60 | 772 | 7.11 | 0.000 |
| R middle occipital gyrus | 36 | -80 | 10 | 796 | 6.93 | 0.000 |
| L middle temporal gyrus | -62 | -46 | -4 | 398 | 7.41 | 0.000 |
| R middle temporal gyrus | 66 | -20 | -8 | 352 | 7.35 | 0.000 |
| L inferior frontal gyrus (pars triangularis) | -50 | 28 | -2 | 175 | 6.41 | 0.000 |
| L hippocampus | -28 | -22 | -18 | 136 | 5.96 | 0.000 |
| R hippocampus | 30 | -22 | -16 | 87 | 5.90 | 0.000 |
| L Amygdala | -22 | -2 | -16 | 86 | 5.75 | 0.000 |
| R Amygdala | 22 | -2 | -16 | 25 | 5.32 | 0.002 |
| attA > attV |  |  |  |  |  |  |
| No activation clusters |  |  |  |  |  |  |
| attV > attA |  |  |  |  |  |  |
| No activation clusters |  |  |  |  |  |  |
|  |  |  |  |  |  |  |

Effect of auditory relative to visual report (repA > repV) and vice-versa (repV > repA); effect of auditory relative to visual attention (attA > attV) and vice-versa (attV > attA). p-values are FWE-corrected at the peak level for multiple comparisons within the entire brain. L: left; R: right.
