# Supplementary material for: Attention controls multisensory perception via two distinct mechanisms at different levels of the cortical hierarchy
Source: PLoS Biol. 2021 Nov 18;19(11):e3001465. doi: 10.1371/journal.pbio.3001465 (PMC8639080; doi:10.1371/journal.pbio.3001465)
Supplement: S7 Table — (a) Across participants’ mean (±SEM) wAV as a function of prestimulus attention (attA, auditory; attV, visual), poststimulus report (repA: auditory; repV: visual) and audiovisual spatial disparity (dispL: low; dispH: high). (b) Across participants’ mean (±SEM) nwAV as a function of prestimulus attention (attA, auditory; attV, visual) and poststimulus report (repA: auditory; repV: visual) for each ROI. A, low-level auditory cortex; aIPS, anterior intraparietal sulcus; hA, higher-order auditory cortex; pIPS, posterior intraparietal sulcus; ROI, region of interest; V1-3, low-level visual cortex. (DOCX) [file pbio.3001465.s011.docx]

**S7 Table. Audiovisual weight index (**$\boldsymbol{w}_{\boldsymbol{AV}}$**) in the psychophysics and fMRI experiments and neural audiovisual weight index (**$\boldsymbol{nw}_{\boldsymbol{AV}}$**) for each region of interest.**

| **a.** $\boldsymbol{w}_{\boldsymbol{AV}}$ **(a.u.)** | attArepA | attVrepA | attArepV | attVrepV |
| --- | --- | --- | --- | --- |
| **Psychophysics** |  |  |  |  |
| dispL | 0.51 (±0.04) | 0.57 (±0.03) | 0.99 (±0.01) | 1.00 (±0.01) |
| dispH | 0.33 (±0.04) | 0.41 (±0.04) | 1.00 (±0.01) | 1.01 (±0.01) |
| **fMRI** |  |  |  |  |
| dispL | 0.43 (±0.06) | 0.51 (±0.05) | 0.99 (±0.01) | 1.01 (±0.01) |
| dispH | 0.24 (±0.04) | 0.33 (±0.05) | 1.00 (±0.01) | 1.01 (±0.01) |
| **b.** $\boldsymbol{nw}_{\boldsymbol{AV}}$ **(a.u.)** | attArepA | attVrepA | attArepV | attVrepV |
| V1-3 | 0.97 (±0.03) | 1.02 (±0.03) | 0.98 (±0.03) | 1.00 (±0.03) |
| pIPS | 0.75 (±0.06) | 0.91 (±0.06) | 0.90 (±0.06) | 0.87 (±0.08) |
| aIPS | 0.55 (±0.07) | 0.69 (±0.07) | 0.78 (±0.06) | 0.76 (±0.06) |
| hA | 0.01 (±0.12) | 0.27 (±0.11) | 0.35 (±0.14) | 0.39 (±0.11) |
| A | 0.30 (±0.16) | -0.01 (±0.17) | 0.27 (±0.18) | 0.40 (±0.19) |

**(a)** Across participants' mean (±SEM) $w_{AV}$ as a function of pre-stimulus attention (attA: auditory; attV: visual), post-stimulus report (repA: auditory; repV: visual) and audiovisual spatial disparity (dispL: low; dispH: high). **(b)** Across participants' mean (±SEM) $nw_{AV}$ as a function of pre-stimulus attention (attA: auditory; attV: visual) and post-stimulus report (repA: auditory; repV: visual) for each ROI. V1-3: low-level visual cortex; pIPS: posterior intraparietal sulcus; aIPS: anterior intraparietal sulcus; hA: higher-order auditory cortex; A: low-level auditory cortex.
