# Supplementary material for: Attention controls multisensory perception via two distinct mechanisms at different levels of the cortical hierarchy
Source: PLoS Biol. 2021 Nov 18;19(11):e3001465. doi: 10.1371/journal.pbio.3001465 (PMC8639080; doi:10.1371/journal.pbio.3001465)
Supplement: S8 Table — Main effects and interactions for (a) behavioural audiovisual weight index (wAV) in the psychophysics and fMRI experiments and (b) neural audiovisual weight index (nwAV) in the 2 (prestimulus attention, Att: attA, attV) × 2 (poststimulus report, Rep: repA, repV) × 2 (audiovisual spatial disparity, Disp: low, high) factorial design. p-Values are based on two-tailed permutation tests apart from those for main effect of prestimulus attention (Att: attV > attA) and poststimulus report (Rep: repV > repA), which are one-tailed because of a priori hypotheses. Effect sizes [95% CI] correspond to the difference of the across participants’ mean empirical effect and the mean of the nonparametric null distribution. A, low-level auditory cortex; aIPS, anterior intraparietal sulcus; hA, higher-order auditory cortex; pIPS, posterior intraparietal sulcus; ROI, region of interest; V1-3, low-level visual cortex. (DOCX) [file pbio.3001465.s012.docx]

**S8 Table.** **Statistical significance (p-value and effect size with 95% CI) of behavioural audiovisual weight index (**$\boldsymbol{w}_{\boldsymbol{AV}}$**) and of neural audiovisual weight index (**$\boldsymbol{nw}_{\boldsymbol{AV}}$**) for each region of interest.**

| **a.** $\boldsymbol{w}_{\boldsymbol{AV}}$ | Att | Rep | Disp | Att×Rep | Att×Disp | Rep×Disp | Att×Rep×Disp |
| --- | --- | --- | --- | --- | --- | --- | --- |
| **Psychophysics** |  |  |  |  |  |  |  |
| p-value (effect size [95% CI]) | .007 (.05 [.02, .07]) | .000 (.54 [.48, .61]) | .000 (.09 [.07, .11]) | .065 (.07 [.03, .12]) | .538 (.01 [-.02, .05]) | .000 (.19 [.16, .23]) | .386 (.03 [-.08, .02]) |
| **fMRI** |  |  |  |  |  |  |  |
| p-value (effect size [95% CI]) | .002 (.05 [.01, .08]) | .000 (.63 [.54, .73]) | .000 (.09 [.07, .11]) | .022 (.07 [.01, .13]) | .705 (.01 [-.02, .03]) | .000 (.19 [.16, .22]) | .404 (.02 [-.08, .03]) |
| **b.** $\boldsymbol{n}\boldsymbol{w}_{\boldsymbol{AV}}$ | Att | Rep | Disp | Att×Rep | Att×Disp | Rep×Disp | Att×Rep×Disp |
| V1-3 | .011 (.03 [0, .06]) | .765 (.01 [-.01, .03]) | .312 (.01 [-.01, .04]) | .288 (.03 [-.02, .08]) | .065 (.04 [0, .08]) | .083 (.04 [0, .08]) | .952 (0  [-.10, .11]) |
| pIPS | .088 (.05 [-.02, .13]) | .130 (.06 [-.04, .15]) | .497 (.02 [-.03, .07]) | .006 (.17 [.07, .28]) | .523 (.04 [-.08, .16]) | .942 (0  [-.11, .10]) | .686 (.08 [-.31, .47]) |
| aIPS | .064 (.06 [-.01, .13]) | .002 (.15 [.07, .23]) | .098 (.07 [0, .14]) | .147 (.18 [-.05, .41]) | .796 (.03 [-.17, .23]) | .919 (0 [-.17, .19]) | .742 (.08 [-.36, .52]) |
| hA | .176 (.10 [-.10, .29]) | .030 (.17 [0, .34]) | .100 (.18 [-.02, .38]) | .329 (.21 [-.19, .60]) | .067 (.29 [0, .57]) | .127 (.38 [-.07, .83]) | .939 (.03 [-.69, .75]) |
| A | .864 (.17 [-.12, .45]) | .335 (.10 [-.36, .56]) | .485 (.15 [-.23, .54]) | .481 (.43 [-.49, 1.36]) | .267 (.44 [.28, 1.16]) | .407 (.50 [-.66, 1.66]) | .941 (.11 [-2.90, 3.11]) |

Main effects and interactions for **(a)** behavioural audiovisual weight index ($w_{AV}$) in the psychophysics and fMRI experiments and **(b)** neural audiovisual weight index (${nw}_{AV}$) in the 2 (pre-stimulus attention, Att: attA, attV) × 2 (post-stimulus report, Rep: repA, repV) × 2 (audiovisual spatial disparity, Disp: low, high) factorial design. P-values are based on two tailed permutation tests apart from those for main effect of pre-stimulus attention (Att: attV > attA) and post-stimulus report (Rep: repV > repA), which are one-sided because of a priori hypotheses. Effect sizes [95% CI] correspond to the difference of the across participants' mean empirical effect and the mean of the non-parametric null-distribution. V1-3: low-level visual cortex; pIPS: posterior intraparietal sulcus; aIPS: anterior intraparietal sulcus; hA: higher-order auditory cortex; A: low-level auditory cortex.
