# Supplementary material for: Attention controls multisensory perception via two distinct mechanisms at different levels of the cortical hierarchy
Source: PLoS Biol. 2021 Nov 18;19(11):e3001465. doi: 10.1371/journal.pbio.3001465 (PMC8639080; doi:10.1371/journal.pbio.3001465)
Supplement: S9 Table — Across participants’ mean (±SEM) as a function of prestimulus attention (attA, auditory; attV, visual), poststimulus report (repA: auditory; repV: visual) and audiovisual spatial disparity (dispN: no disparity; dispL: low; dispH: high). (DOCX) [file pbio.3001465.s013.docx]

**S9 Table. Proportion of correct responses in the psychophysics and fMRI experiments.**

| **Proportion correct responses** | attArepA | attVrepA | attArepV | attVrepV |
| --- | --- | --- | --- | --- |
| **Psychophysics** |  |  |  |  |
| dispN | 0.93 (±0.01) | 0.91 (±0.01) | 0.99 (±0.01) | 0.99 (±0.01) |
| dispL | 0.45 (±0.04) | 0.39 (±0.03) | 0.96 (±0.01) | 0.98 (±0.01) |
| dispH | 0.56 (±0.04) | 0.45 (±0.04) | 0.97 (±0.01) | 0.99 (±0.01) |
| **fMRI** |  |  |  |  |
| dispN | 0.92 (±0.01) | 0.90 (±0.02) | 0.99 (±0.01) | 0.99 (±0.01) |
| dispL | 0.52 (±0.05) | 0.45 (±0.05) | 0.96 (±0.01) | 0.98 (±0.01) |
| dispH | 0.62 (±0.05) | 0.51 (±0.05) | 0.97 (±0.01) | 0.98 (±0.01) |

Across participants' mean (±SEM) as a function of pre-stimulus attention (attA: auditory; attV: visual), post-stimulus report (repA: auditory; repV: visual) and audiovisual spatial disparity (dispN: no disparity; dispL: low; dispH: high).
