# Supplementary material for: Attention controls multisensory perception via two distinct mechanisms at different levels of the cortical hierarchy
Source: PLoS Biol. 2021 Nov 18;19(11):e3001465. doi: 10.1371/journal.pbio.3001465 (PMC8639080; doi:10.1371/journal.pbio.3001465)
Supplement: S10 Table — Using Bayesian model comparison, we assessed the influence of prestimulus attention and poststimulus report in a 2 × 3 factorial model space. Along the first factor, we assessed the influence of prestimulus attention comparing models in which the auditory and visual variances were (i) constant (Att fixed: σAattA2 = σAattV2, σVattA2 = σVattV2); or (ii) different (Att free: σAattA2, σAattV2, σVattA2, σVattV2) across prestimulus attention. Along the second factor, we assessed the influence of poststimulus report by comparing (i) an FF model in which the sensory variances were fixed (Rep fixed: σArepA2 = σArepV2, σVrepA2 = σVrepV2); (ii) an FF model in which the sensory variances were allowed to differ between auditory and visual report (Rep free: σArepA2, σArepV2, σVrepA2, σVrepV2); and (iii) a BCI model in which the influence of poststimulus report arises via a late flexible readout. We report across participants’ mean (±SEM) of the models parameters: Pcommon, prior common source probability; σP, spatial prior standard deviation (° visual angle); σA, auditory likelihood standard deviation (° visual angle); σV, visual likelihood standard deviation (° visual angle). In addition, R2, coefficient of determination; relBIC, BIC of a model summed over participants (BIC = LL − 0.5 × P × ln(N), LL = log-likelihood, P = number of parameters, N = number of data points) relative to the “BCI Att free” model (a model with smaller relBIC provides better data explanation); pEP, protected exceedance probability (probability that a model is more likely than the other models, beyond differences due to chance). BCI, Bayesian causal inference; FF, forced fusion. (DOCX) [file pbio.3001465.s014.docx]

**S10 Table. Bayesian modelling results in the psychophysics and fMRI experiments.**

| **Model** | $P_{common}$ | $\sigma_{P}$ | $\sigma_{A}$ | $\sigma_{V}$ | $R^{2}$ | $relBIC$ | $pEP$ |
| --- | --- | --- | --- | --- | --- | --- | --- |
| **Psychophysics** | |  |  |  |  |  |  |
| FF  Att fixed  Rep fixed | n/a | 28.67  (±0.84) | 10.31  (±0.50) | 6.00  (±0.21) | 0.61 (±0.01) | 5,136.52 | 0 |
| FF  Att free  Rep fixed | n/a | 28.32 (±0.83) | attA: 11.44 (±0.92)  attV:  12.53 (±1.24) | attA: 6.37 (±0.21)  attV:  5.68 (±0.27) | 0.62 (±0.01) | 5,228.35 | 0 |
| FF Att fixed  Rep free | n/a | 26.82  (±1.28) | repA:  7.31  (±0.46)  repV:  22.78  (±1.41) | repA:  9.40  (±0.93)  repV:  1.77  (±0.12) | 0.83 (±0.01) | 850.54 | 0 |
| FF  Att free  Rep free | n/a | 20.25  (±1.39) | attArepA:  13.84  (±1.90)  attArepV:  22.56  (±1.25)  attVrepA:  13.42  (±1.70)  attVrepV:  20.62  (±1.72) | attArepA:  19.78  (±1.64)  attArepV:  1.99  (±0.14)  attVrepA:  15.72  (±1.87)  attVrepV:  1.57  (±0.11) | 0.83 (±0.01) | 1,423.16 | 0 |
| BCI  Att fixed | 0.44  (±0.04) | 62.25  (±11.97) | 7.95  (±0.57) | 0.13  (±0.03) | 0.83 (±0.01) | 765.25 | 0 |
| BCI  Att free | 0.62 (±0.03) | 22.09 (±1.85) | attA:  6.89 (±0.49)  attV:  8.70 (±0.72) | attA:  1.94 (±0.11)  attV:  1.57 (±0.12) | 0.87 (±0.01) | 0 | 1 |
| **fMRI** |  |  |  |  |  |  |  |
| FF model  Att fixed  Rep fixed | n/a | 29.61  (±0.20) | 9.48  (±0.36) | 6.33  (±0.35) | 0.58 (±0.02) | 1,241.04 | 2×10^-4^ |
| FF model  Att free  Rep fixed | n/a | 29.72 (±0.14) | attA:  9.33 (±0.36)  attV:  11.39 (±1.74) | attA:  6.76 (±0.39)  attV:  5.91 (±0.42) | 0.58 (±0.02) | 1,238.03 | 2×10^-4^ |
| FF model Att fixed  Rep free | n/a | 24.98  (±2.31) | repA:  6.31  (±0.44)  repV:  15.96  (±1.74) | repA:  9.14  (±0.89)  repV:  1.92  (±0.24) | 0.83 (±0.02) | 1,404.26 | 2×10^-4^ |
| FF model  Att free  Rep free | n/a | 17.96  (±2.00) | attArepA:  8.25  (±2.00)  attArepV:  24.30  (±2.24)  attVrepA:  11.60  (±2.42)  attVrepV:  24.22  (±1.97) | attArepA:  17.59  (±2.27)  attArepV:  2.18  (±0.18)  attVrepA:  18.14  (±2.54)  attVrepV:  1.77  (±0.27) | 0.82 (±0.02) | 2,121.08 | 2×10^-4^ |
| BCI  Att fixed | 0.54  (±0.05) | 21.16  (±3.00) | 6.57  (±0.56) | 2.07  (±0.18) | 0.85 (±0.02) | 1,582.27 | 1.7×10^-4^ |
| BCI  Att free | 0.53 (±0.05) | 19.15 (±2.64) | attA:  6.09 (±0.55)  attV:  7.43 (±0.73) | attA:  2.15 (±0.18)  attV:  1.69 (±0.28) | 0.85 (±0.02) | 0 | 0.99 |

Using Bayesian model comparison, we assessed the influence of pre-stimulus attention and post-stimulus report in a 2 × 3 factorial model space. Along the first factor, we assessed the influence of pre-stimulus attention comparing models in which the auditory and visual variances were i. constant (Att fixed: $\sigma_{A attA}^{2}$ = $\sigma_{A attV}^{2}$, $\sigma_{V attA}^{2}$ = $\sigma_{V attV}^{2}$) or ii. different (Att free: $\sigma_{A attA}^{2}$, $\sigma_{A attV}^{2}$, $\sigma_{V attA}^{2}$, $\sigma_{V attV}^{2}$) across pre-stimulus attention. Along the second factor, we assessed the influence of post-stimulus report by comparing i. a forced fusion (FF) model in which the sensory variances were fixed (Rep fixed: $\sigma_{A repA}^{2}$ = $\sigma_{A repV}^{2}$, $\sigma_{V repA}^{2}$ = $\sigma_{V repV}^{2}$), ii. a forced fusion model in which the sensory variances were allowed to differ between auditory and visual report (Rep free: $\sigma_{A repA}^{2}$, $\sigma_{A repV}^{2}$, $\sigma_{V repA}^{2}$, $\sigma_{V repV}^{2}$), and iii. a Bayesian Causal Inference (BCI) model in which the influence of post-stimulus report arises via a late flexible read-out. We report across participants' mean (±SEM) of the models parameters: $P_{common}$, prior common-source probability; $\sigma_{P}$, spatial prior standard deviation (° visual angle); $\sigma_{A}$, auditory likelihood standard deviation (° visual angle); $\sigma_{V}$, visual likelihood standard deviation (° visual angle). In addition: $R^{2}$, coefficient of determination; $relBIC$, Bayesian information criterion of a model summed over subjects (BIC = LL − 0.5 × P × ln(N), LL = log-likelihood, P = number of parameters, N = number of data points) relative to the BCI Att free model (a model with smaller relBIC provides better data explanation); $pEP$, protected exceedance probability (probability that a model is more likely than the other models, beyond differences due to chance).
