# Supplementary material for: Attention controls multisensory perception via two distinct mechanisms at different levels of the cortical hierarchy
Source: PLoS Biol. 2021 Nov 18;19(11):e3001465. doi: 10.1371/journal.pbio.3001465 (PMC8639080; doi:10.1371/journal.pbio.3001465)
Supplement: S11 Table — Effect of auditory localisation collapsing across spatial locations (Task > Baseline) and separately for left versus right lateralised sounds (SoundL > SoundR; SoundR > SoundL). p-Values are FWE corrected at the peak level for multiple comparisons within the entire brain. FWE, family-wise error; L, left; R, right. (DOCX) [file pbio.3001465.s015.docx]

**S11 Table. fMRI results of unisensory auditory localisation inside the scanner.**

| Brain regions | MNI coordinates (mm) | | | Cluster size (voxels) | z-score (peak) | p _FWE-corrected_ (peak) |
| --- | --- | --- | --- | --- | --- | --- |
|  | x | y | z |  |  |  |
| Task > Baseline |  |  |  |  |  |  |
| R cerebellum | 18 | -52 | -24 | 618 | 7.47 | 0.000 |
| L planum temporale | -50 | -30 | 10 | 489 | 7.29 | 0.000 |
| R planum temporale | 64 | -34 | 16 | 114 | 6.42 | 0.000 |
| R parietal operculum | 64 | -26 | 22 |  | 5.55 | 0.000 |
| L post-central gyrus | -36 | -28 | 50 | 795 | 6.72 | 0.000 |
| L pre-central gyrus | -36 | -12 | 64 |  | 6.42 | 0.000 |
| R superior frontal gyrus | 26 | -16 | 46 | 79 | 6.25 | 0.000 |
| L superior frontal gyrus | -22 | -16 | 52 | 120 | 6.22 | 0.000 |
|  |  |  |  |  |  |  |
| SoundL > SoundR |  |  |  |  |  |  |
| R pre-central gyrus | 26 | -16 | 74 | 54 | 6.34 | 0.000 |
| R planum polare | 34 | -22 | 6 | 61 | 5.95 | 0.000 |
| R parietal operculum | 52 | -24 | 22 | 29 | 5.75 | 0.001 |
| R planum temporale | 50 | -32 | 18 |  | 4.89 | 0.031 |
| R planum temporale | 52 | -22 | 4 | 40 | 5.74 | 0.001 |
| R superior parietal lobule | 22 | -52 | 72 | 76 | 5.72 | 0.001 |
|  |  |  |  |  |  |  |
| SoundR > SoundL |  |  |  |  |  |  |
| R inferior frontal gyrus (pars opercularis) | 52 | 20 | 28 | 288 | 7.13 | 0.000 |
| L post-central gyrus | -36 | -28 | 52 | 654 | 7.03 | 0.000 |
| L planum temporale | -50 | -32 | 8 | 143 | 6.30 | 0.000 |
| L parietal operculum | -60 | -36 | 14 |  | 5.97 | 0.000 |
| L precuneus | -8 | -74 | 38 | 48 | 5.79 | 0.000 |
| L superior parietal lobule | -38 | -54 | 58 | 35 | 5.69 | 0.001 |
| R superior parietal lobule | 38 | -68 | 48 | 65 | 5.69 | 0.001 |
|  |  |  |  |  |  |  |

Effect of auditory localisation collapsing across spatial locations (Task > Baseline) and separately for left versus right lateralised sounds (SoundL > SoundR; SoundR > SoundL). p-values are FWE-corrected at the peak level for multiple comparisons within the entire brain. L: left; R: right.
