# Supplementary figures and images for: Attention controls multisensory perception via two distinct mechanisms at different levels of the cortical hierarchy
Source: PLoS Biol. 2021 Nov 18;19(11):e3001465. doi: 10.1371/journal.pbio.3001465 (PMC8639080; doi:10.1371/journal.pbio.3001465)

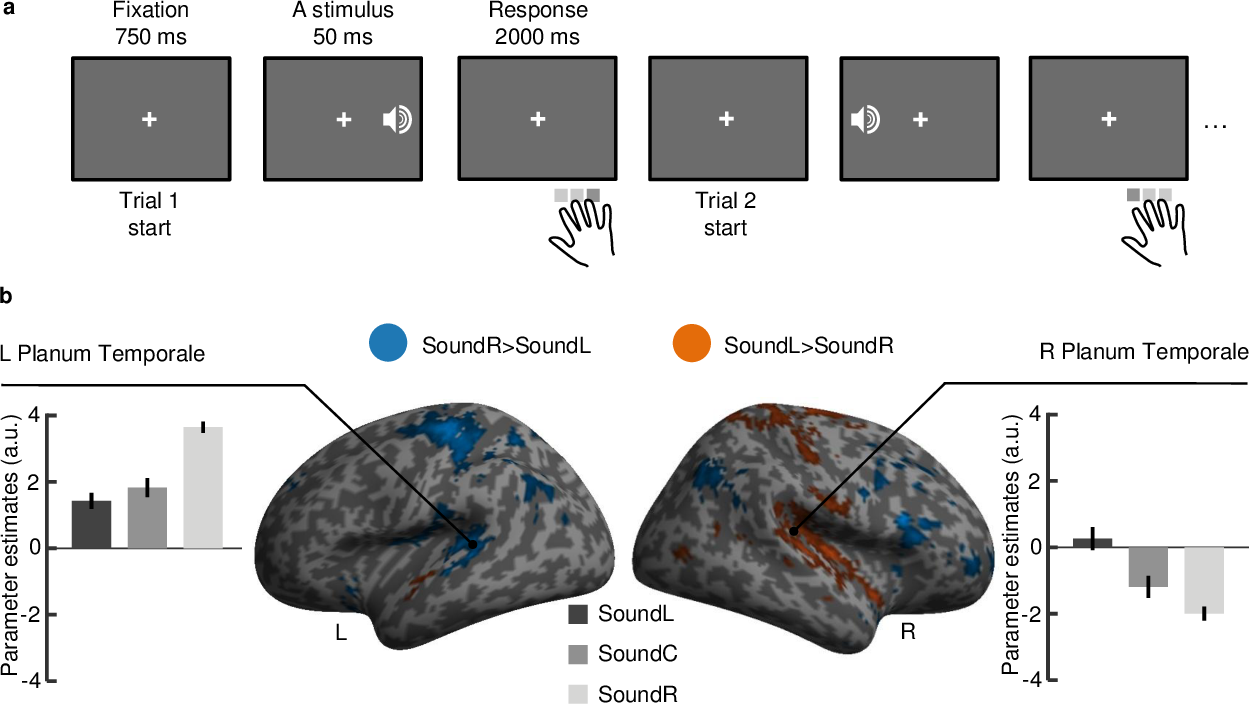

Supplement: S1 Fig — (a) Experimental procedure: after each sound presentation, participants reported their perceived auditory location via button press with the correspondent key. (b) Increases of BOLD response for lateralised right versus left sounds (blue) and vice versa (orange) are rendered on an inflated canonical brain (p < 0.001 uncorrected at peak level for visualisation purposes, extent threshold k > 0 voxels). Bar plots represent across participants’ mean (±SEM) parameter estimates in nondimensional units (corresponding to percentage whole-brain mean) from left (x = −50, y = −32, z = 8) and right (x = 52, y = −22, z = 4) plana temporalia. The data used to make this figure are available in S3 Data. C, centre; L, left; R, right. (TIF) [file pbio.3001465.s016.tif]

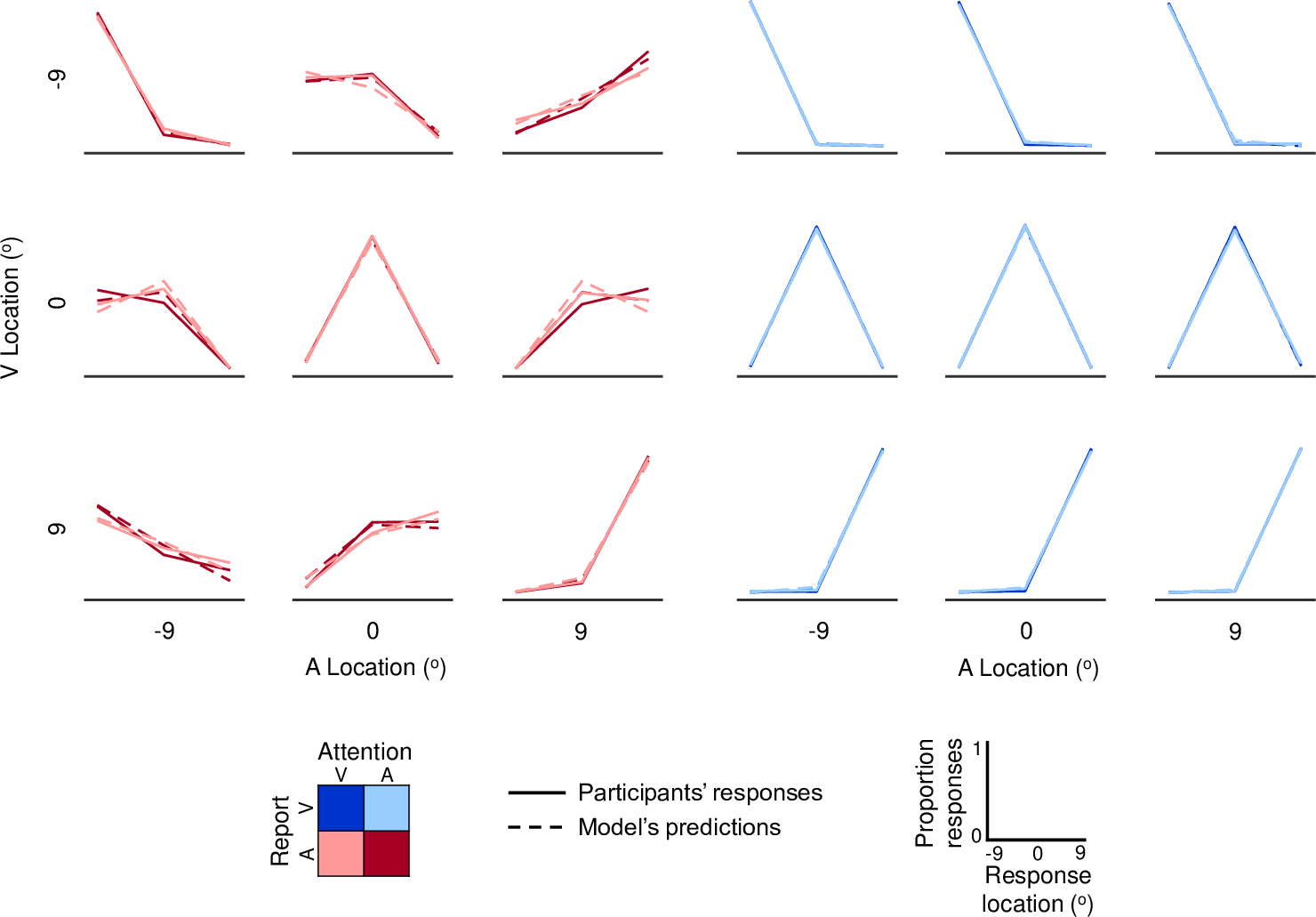

Supplement: S2 Fig — The distribution of spatial estimates (across participants’ mean) given by observers’ behavioural localisation responses (solid lines) or predicted by the BCI model with attentional effects (i.e., “BCI model, Att free”) fitted to observers’ behavioural responses (dashed lines) are shown across all conditions in our a 3 (auditory location) × 3 (visual location) × 2 (prestimulus attention: auditory, visual) × 2 (poststimulus report: auditory, visual) factorial design. The data used to make this figure are available in S2 Data. BCI, Bayesian causal inference. (TIF) [file pbio.3001465.s017.tif]
